# Trans-Ancestry Mutation Landscape of Hepatoblastoma Genomes in Children

**DOI:** 10.3389/fonc.2021.669560

**Published:** 2021-04-21

**Authors:** Jie Liu, Chengwen Gao, Liping Wang, Xuemin Jian, Mingdi Ma, Tong Li, XiWei Hao, Qian Zhang, Yuanbin Chen, Jing Zhao, Haitao Niu, Chengzhan Zhu, Jie Zhao, Nan Xia, Zhiqiang Li, Qian Dong

**Affiliations:** ^1^Department of Pediatric Surgery, Affiliated Hospital of Qingdao University, Qingdao University, Qingdao, China; ^2^Department of Pediatric Surgery, Yijishan Hospital of Wannan Medical College, Wannan Medical College, Wuhu, China; ^3^Laboratory of Medical Biology, Medical Research Center, The Affiliated Hospital of Qingdao University & The Biomedical Sciences Institute of Qingdao University (Qingdao Branch of SJTU Bio-X Institutes), Qingdao University, Qingdao, China; ^4^Key Laboratory, Department of Urology and Andrology, Medical Research Center, The Affiliated Hospital of Qingdao University, Qingdao University, Qingdao, China; ^5^Bio-X Institutes, Key Laboratory for the Genetics of Developmental and Neuropsychiatric Disorders (Ministry of Education) and the Collaborative Innovation Center for Brain Science, Shanghai Jiao Tong University, Shanghai, China; ^6^Department of Urology, The Affiliated Hospital of Qingdao University, Qingdao University, Qingdao, China; ^7^Department of Hepatobiliary and Pancreatic Surgery, The Affiliated Hospital of Qingdao University, Qingdao, China; ^8^Institute of Digital Medicine and Computer-assisted Surgery, Qingdao University, Qingdao, China; ^9^Shandong Provincial Key Laboratory of Digital Medicine and Computer-assisted Surgery, Qingdao University, Qingdao, China; ^10^Shandong College Collaborative Innovation Center of Digital Medicine Clinical Treatment and Nutrition Health, Qingdao University, Qingdao, China

**Keywords:** hepatoblastoma, children, whole-exome sequencing, somatic mutation, landscape

## Abstract

Hepatoblastoma (HB) is the most common malignant tumor in the liver of infants and young children. The incidence rate varies among different populations. However, genetic differences in HB patients with different epidemiological and ancestral backgrounds have not been found. In this study, we aim to analyze data from 16 patients treated at our center and collected published data from whole-exome sequencing studies on HB, and to explore the genetic differences between races. Data from a total of 75 HB patients of three races (24 Asian, 37 Caucasian and 14 Hispanic) were analyzed. We identified 16 genes with recurrent somatic mutations and 7 core pathway modules. Among them, the Wnt/β-catenin pathway had the highest mutation rate, and the mutation frequency in Caucasians and Hispanics was approximately twice as high as that in Asians. In addition, this study compared the characteristics of gene mutations between patients who underwent preoperative chemotherapy and those who did not and found that there was no significant difference in gene mutations between the two groups. We also preliminarily verified the function of cancer-associated candidate genes (CTNNB1 and KMT2D). In conclusion, we found ethnic differences in HB biology at the genomic level, which expands our understanding of the genetics of HB in children.

## Introduction

Hepatoblastoma (HB) is the most common malignant tumor in the liver of infants and young children, accounting for approximately 80% of liver tumors in children (1, 2). The HB onset usually occurs in children younger than 3 years old and rarely in those older than 5 years old (3). The incidence rate of children’s HB is increasing and varies between ancestral and geographic regions (4, 5). Studies have reported that the incidence rate of HB in Asians is higher than that in Europeans (5, 6). At present, it is not clear whether there are differences in the gene mutation landscape of HB between ancestors. Complete tumor resection is the main method for the treatment of HB. However, many children with HB are ineligible for resection at the initial stage of diagnosis because the tumor volume is too large or the tumor is too close to main blood vessels (7). With the application of platinum-based neoadjuvant chemotherapy, a large number of patients who could not initially undergo surgery have achieved complete tumor resection by reducing the tumor volume before surgery, and the prognosis of children with this disease has been greatly improved (8). However, studies have found that some tumors may develop drug resistance as the number of chemotherapy cycles increases (9). Whether there is a significant difference between the gene mutations in patients who undergo chemotherapy before surgery and those in patients who do not remains unclear but is very important for studying the mechanism of drug resistance. Unfortunately, due to the lack of sequencing data from tumor samples collected after preoperative chemotherapy, no related studies have been reported.

In recent years, with the emergence of next-generation sequencing technology, some scholars have carried out whole-exome sequencing (WES) of HB and identified some genes with significant mutations (10–13). These advances now make it possible to compare tumor mutations by ancestry and may provide some genomic evidence for targeted therapy research in different ancestral groups (14, 15). Here, we collected WES data from 16 cases of children’s HB treated at our center and integrated four databases of HB sequencing data published by other scholars worldwide for analysis. To the best of our knowledge, this study represents the genome landscape of 75 HB patients from a large ancestrally diverse population (24 Asian, 37 Caucasian and 14 Hispanic patients). Herein, we analyzed impact of chemotherapy intervention. In addition, the identification of genes with recurrent mutations in these genomic data may help us to detect novel cancer-associated genes.

## Materials and Methods

### Data Collection

**Figure 1:**
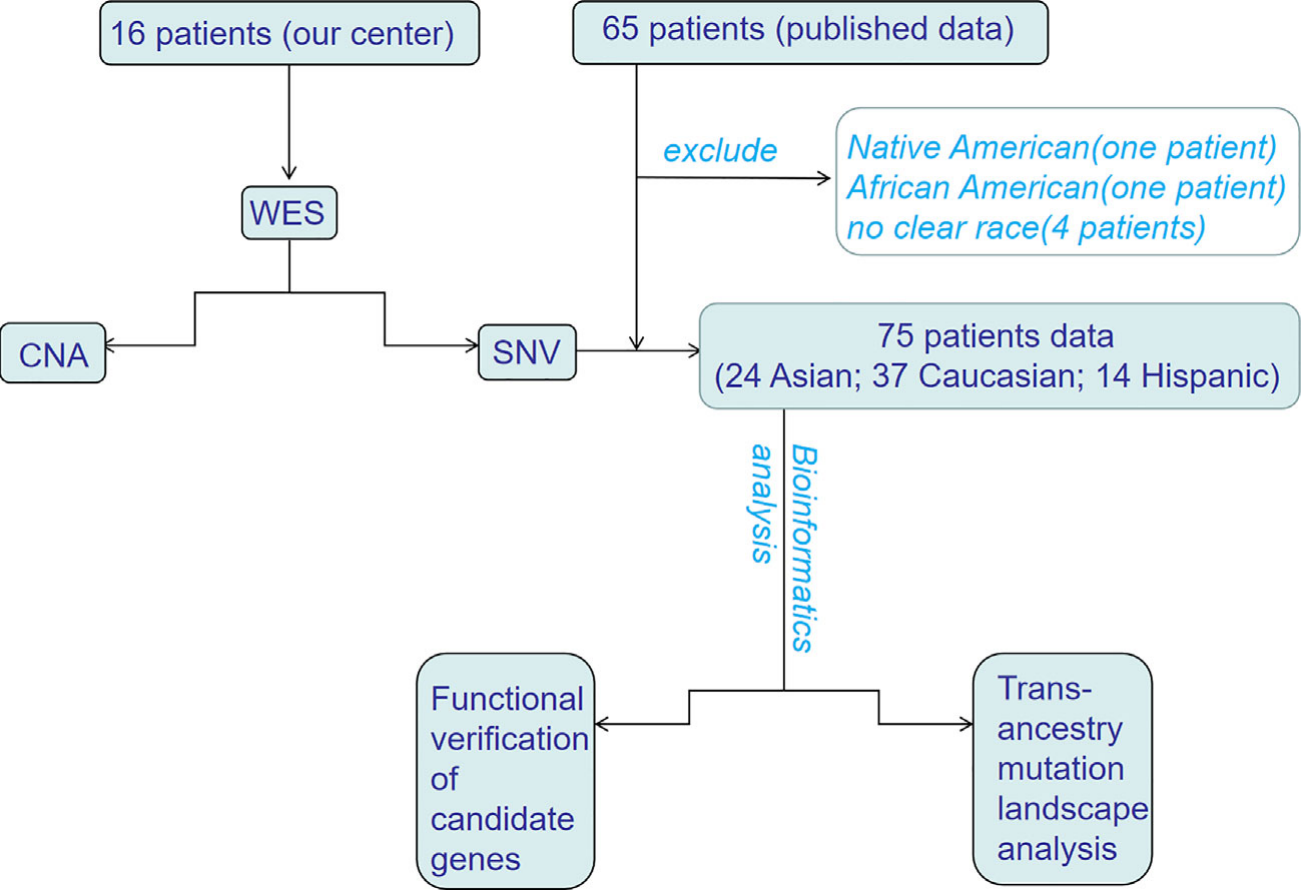
The flowchart of trans-ancestry WES of hepatoblastoma. WES, whole-exome sequencing; SNV, single nucleotide variant; CNA, copy number alteration.

In this study, we performed WES of 16 patients with HB at the Affiliated Hospital of Qingdao University and analyzed the data. This study was approved by the Ethics Committee of the Affiliated Hospital of Qingdao University (No. QYFY-WZLL-25777), and written informed consent for the use of specimens was obtained from each patient or a family member. In addition, data from four published studies on WES of HB were also integrated. To reduce the analysis bias caused the limited number of samples, samples with unknown pedigrees or ethnic groups with fewer than two people were not included. Since the current research is mainly focused on China, the United States, Germany and Brazil, patients were divided into three ethnic groups: Asian, Caucasian, and Hispanic (**Figure 1**). All samples were assessed by FastQC (http://www.bioinformatics.babraham.ac.uk/projects/fastqc/) for quality control.

### Sample Selection and Preparation (16 Patients in Our Center)

Sixteen children (0-14 years) with HB who received treatment at our center between July 2017 and March 2019 were selected. The clinical details of the patients are shown in **Supplementary Table 1**. All patients were diagnosed with HB by fine-needle biopsy before operation or chemotherapy. We evaluated the feasibility of one-stage surgical resection according to the size of the tumor and the distance from the main blood vessels before operation. If the tumor cannot be resected in one-stage, we need preoperative chemotherapy. Detailed methods were described in our prior paper (7). Formalin-fixed paraffin-embedded (FFPE) samples of tumors and corresponding noncancerous liver tissue were obtained. All samples were evaluated by a senior child liver tumor pathologist, and then 20 6-μm thick sections were prepared for DNA extraction.

### DNA Extraction

The MagPure FFPE DNA LQ (Magen, China) Kit was used to extract DNA from FFPE samples according to the manufacturer’s instructions. A Qubit fluorometer and agarose gel electrophoresis were used to detect the concentration, integrity and purification of samples.

### Library Construction and WES

According to the manufacturer’s instructions, the DNA of each sample was sequenced on the BGISEQ-500 platform (BGI Shenzhen, China) (16). After reading the sequence, we obtained the raw sequencing data of 32 samples from 16 patients and stored them in FASTQ format.

### Identification and Validation of Somatic Mutations

Raw reads from each library were mapped to the reference human genome (NCBI build 38, hg 38) using a Burrows Wheeler Alignment tool (BWA) v 0.7.17 (17, 18), and the outputs were locally realigned using the Indel Realignment tool of the Genome Analysis Toolkit (GATK) v4.1.2.0 (19). After local realignment, recalibration was performed by the Base Recalibrator tool from GATK. Somatic mutations were detected using MuTect v2 (20) and Varscan2. Variant filtration was performed according to the NCI’s Genomic Data Commons (GDC) workflows. Variant annotation was performed using Oncotator (21) and Variant Effect Predictor (VEP) (22).

Sanger sequencing was used to validate recurrent somatic mutations. The gene bank exon sequence of the corresponding gene was found through http://www.ncbi.nih.gov/, and the gene tool software was used to design the primers for each candidate mutation region. The genomic DNA was used as the template to amplify the target DNA fragment. After purification and identification, the PCR product was added to BigDye ^®^ Terminator v3.1 (ABI) for PCR amplification and operation. Sequencing, comparison with the mRNA standard sequence, and identification of mutation sites was performed with the GenBank database.

### Copy Number Alteration (CNA) Analysis

CNAs in normal samples were compared to matching tumor samples using a relative coverage method performed in GATK v4.1.2.0. The hidden Markov model (HMM) algorithm is used to detect the CNA region. Significant CNA was judged by the following criteria: copy number greater than 2.5 or less than 1.5, indicating the gain or loss of genomic content, respectively; and a minimum physical length of the putative CNA of more than 100 kb. The Genetic Identification of Significant Targets in Cancer version 2.0 (GISTIC2.0) program was used to identify genes affected by the somatic CNA across all the samples.

### Statistics of Single Gene Mutation Frequency

The number of mutations per patient and the number of patients with at least one mutation in each gene were calculated. The percentage of cases with at least one Tier 1 single nucleotide variant (SNV) in each gene was calculated as the mutation frequency of the gene. The types of mutations included synonymous, nonsynonymous, frameshift deletion, frameshift insertion, missense, and nonsense variants. In addition, the number of somatic mutations per chromosome was calculated. The relationship between age and somatic mutations was analyzed using a linear regression model. To avoid age-related biases, a Hispanic patient over age 14 (HB28, 17) was excluded from the analysis because most of the patients were under 14 years old (HB in patients over 14 years old in some countries or regions is treated surgically in the adult liver center). Cluster analysis was used to identify recurrent somatic mutations, which include not only the mutation of the same gene in two or more patients but also the mutation of two or more loci in the same patient.

### Analysis of Recurrent Somatic Mutations Affecting Biological Processes

Functional analysis of recurrent somatic mutations was performed by DAVID (https://david.ncifcrf.gov/). The Gene Oncology (GO) database was used to analyze whether there are any functional genetic changes in HB and whether the mutation frequency differed among races. The Entrez ID of each gene was obtained from the Gene2GO file of NCBI (https://www.ncbi.nlm.nih.gov/) and mapped to GO terms.

### Pathway Enrichment Analysis

All the collected data sets were subjected to Kyoto Encyclopedia of Genes and Genomes (KEGG) pathway enrichment analysis and compared with The Cancer Genome Atlas (TCGA) database (https://tcga-data.nci.nih.gov/tcga) to annotate the gene function, and the mutation rate of key genes in each race was calculated.

### Analysis of Genes With Somatic Mutations in Preoperative Chemotherapy Group (CG) and Nonchemotherapy Group (NCG) Patients

According to the clinical information of patients in the data set, the patients were divided into two groups: the preoperative CG and the NCG. The genes with recurrent somatic mutations and their enriched pathways were analyzed in the same way as the abovementioned ethnic classification analysis.

### Functional Verification of Candidate Cancer-Associated Genes

*CTNNB1* and *KMT2D*, the two genes with the most frequent recurrent mutations in the sequencing data of our center, are also the top two genes in terms of mutation frequency in the three ethnic groups. We selected 20 pairs of HB tumors (including 16 cases of sequenced tumors) and paracancerous liver tissues to carry out immunohistochemical (IHC) staining for the proteins expressed by the two genes (β-catenin and KMT2D). The experiment was carried out as described by our previous study (7). The tissues were incubated with antibodies against β-catenin (ab16051, 1:100; Abcam, USA) and KMT2D (ab224156, 1:200, Abcam, USA) for 1 hour at room temperature and then incubated with secondary antibody (Dako); sections were stained with hematoxylin. The staining score was determined according to the percentage of positively stained cells (0 = less than 5%, 1 = 5-25%, 2 = 26-50%, 3 = 51-75%, 4 = more than 75%, positive cells) and the intensity of staining (0-3). The total score was obtained by multiplying the percentage score by the intensity score.

### Cell Lines and Lentivirus Infection

Two human hepatoblastoma cell lines, HUH-6 and HepG2, were obtained from Wuhan Procell Life Science & Technology Co,.Ltd. (Wuhan, China). They were cultured in DMEM medium supplemented with 10% fetal bovine serum, 100 U/ml penicillin and 100 μg/ml streptomycin, and cultured at 37°C and 5% CO2. These cells were regularly shown to be free from mycoplasma contamination. Knockdown lentiviruses for *CTNNB1* and *KMT2D* as well as control lentivirus were purchased from Shanghai Genechem Co.,Ltd (Shanghai, China) and details of the shRNA information were as follows: *CTNNB1*-shRNA: sense (5’-3’): GGAUGUGGAUACCUCCCAATT, antisense (5’-3’): UUGGGAGGUAUCCACAUCCTT; *KMT2D*-shRNA: sense (5’-3’): GAGCACATGGAGTGGAAATT, antisense (5’-3’): AATTTCGCACTCCATGTGCTC; The HUH-6 and HepG2 cells were infected by the lentivirus according to the manufacturer’s instructions and then selected with 2 µg/ml puromycin (MedChemExpress, Monmouth Junction, NJ, USA).

### qRT-PCR

Total RNA from HUH-6 and HepG2 cells were extracted by TRIzol (Takara, Kusatsu, Japan), then 1000 ng of total RNA was transcribed into cDNA using a PrimeScript™ RT reagent kit (Takara). The cDNA was used as a template with SYBR Green PCR master mix (Takara), and the primers for *CTNNB1*, *KMT2D* and GAPDH were as follows: *CTNNB1* (forward): 5’-GGGATTTTCTCAGTCCTTCAC-3’; *CTNNB1* (reverse): 5’-GTCCTCGTCATTTAGCAGTTT-3’; *KMT2D* (forward): 5’-TCACCTGTGCTCTATGCCAACA-3’; *KMT2D* (reverse): 5’-TCGGTCAGTCTTACGGGCTATG-3’; GAPDH (forward): 5’-CAGGGCTGCTTTTAACTCTGGTA-3’; and GAPDH (reverse): 5’-CATGGGTGGAATCATATTGGAAC-3’. All primers were purchased from Shanghai Genechem Co.,Ltd. Reactions were carried out using the AriaMx real‐time PCR system and analyzed using the AriaMx software v1.1 (Agilent Technologies, Santa Clara, CA, USA). The fold changes in the expression of each gene were calculated by the comparative thresholdcycle (Ct) method using the formula 2−(ΔΔCt).

### Western Blotting

Total protein was extracted from HUH-6 and HepG2 cells in SDS buffer, and the protein concentration was measured by bicinchoninic acid kit (Thermo Fisher Scientific, Waltham, MA, USA). Afterwards, the proteins (30 μg) were separated by 10% SDS-polyacrylamide gel electrophoresis and then transferred to polyvinylidene fluoride membranes (Millipore, Billerica, USA). After blocking for 1 h in 5% fat-free milk, the membranes were incubated with primary antibody at 4°C overnight, using antibodies for CTNNB1 (Cat: E-AB-22111, Elabscience, Wuhan, China, 1: 1000 dilution). Then, the membranes were incubated with horseradish peroxidase conjugated secondary antibodies (Jackson ImmunoResearch, West Grove, PA, USA, 1:10000 dilution) for another 1 h at room temperature. The immune complexes were detected using an enhanced chemiluminescence kit (Millipore). The results were normalized using GAPDH to correct for differences in protein loading. Densitometric analysis was conducted using AlphaView SA software.

### MTT Assay

HUH-6 and HepG2 cells (1000 or 2000 cells/well) were seeded in 96-well plates and incubated in 200 μl of DMEM or MEM with 10% FBS. Subsequently, on the indicated day, 10 μl of MTT solution (5 mg/ml) was added to each well, and the plates were incubated for another 4 h at 37°C. Then the MTT formazan precipitate was dissolved in DMSO and was measured at the absorbance of 490 nm using a microplate reader (Thermo).

### Colony Formation

HUH-6 and HepG2 cells (500 or 1000 cells/well) were seeded in 6-well plates and cultured at 37°C for 2 weeks. Subsequently, the cells were washed with PBS three times and fixed with 4% polyformaldehyde for 15 min. Then the cells were stained with 0.1% crystal violet for 15 min and washed by distilled water. Megascopic cell colonies were counted by Image J and the results were statistically analyzed by GraphPad.

### Statistical Analysis

Each experiment was performed at least three times and the error bars represent the standard error of the mean. Statistical analyses were performed using Graph Prism 6.0 software (GraphPad, La Jolla, CA, USA). Comparisons between two groups were calculated using unpaired t-tests, and values of P<0.05 were considered statistically significant (*P<0.05, **P<0.01, ***P<0.001, ****P<0.0001).

## Results

### WES of HB

**Figure 2:**
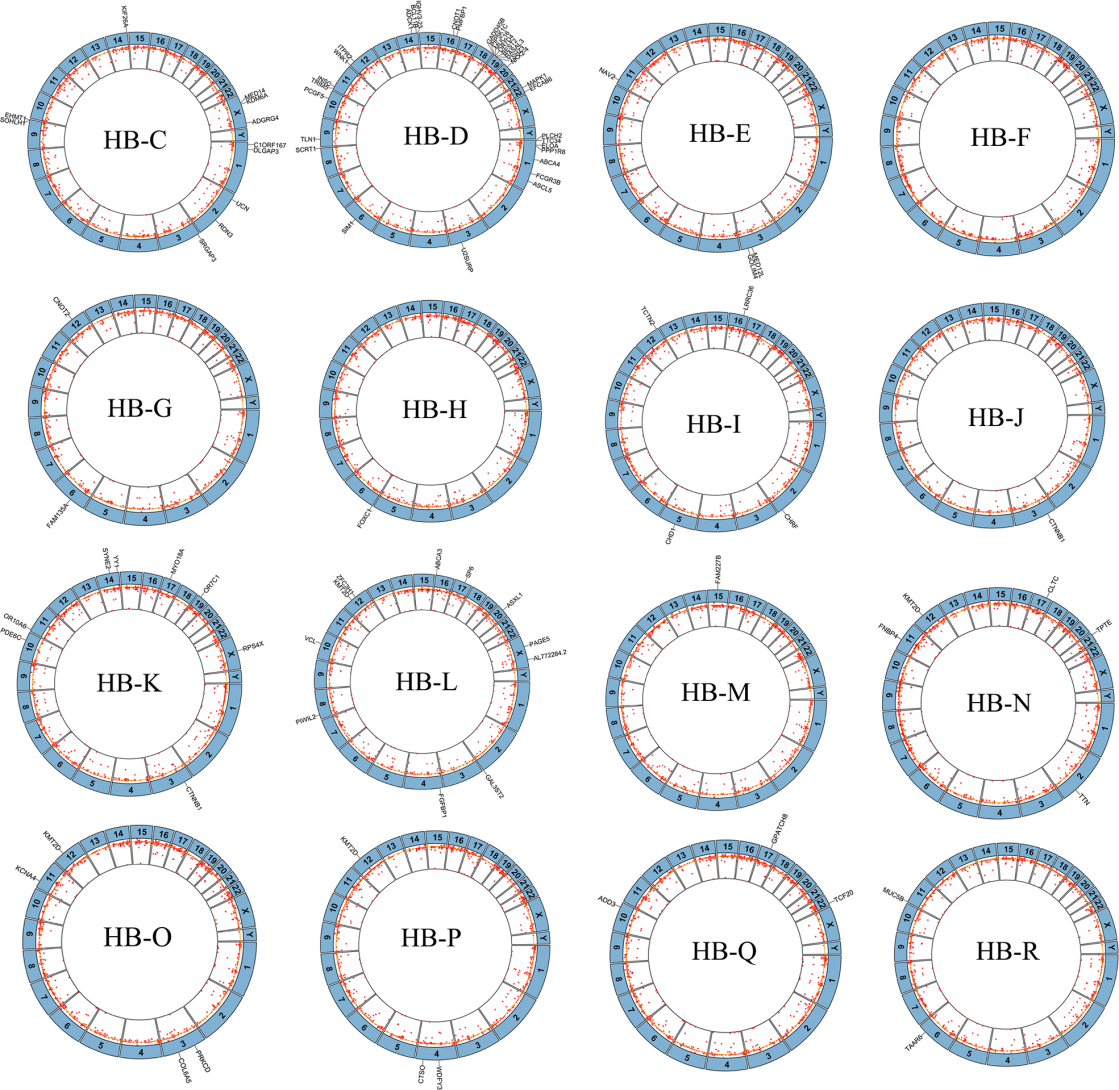
Mutations and copy number alterations (CNAs) in hepatoblastoma tumors in our center. Circos plots of sequence alterations and DNA CNAs in each of the 16 hepatoblastoma samples, with mutated genes depicted outside of each circle. The inside ring shows copy number gains (outside yellow circle) and losses (inside yellow circle). Non-chemotherapy group HB patients: HB-K, HB-L, HB-M, HB-N, HB-P, HB-Q, HB-R. Chemotherapy group HB patients: HB-C, HB-D, HB-E, HB-F, HB-G, HB-H, HB-I, HB-J, HB-O.

**Figure 3:**
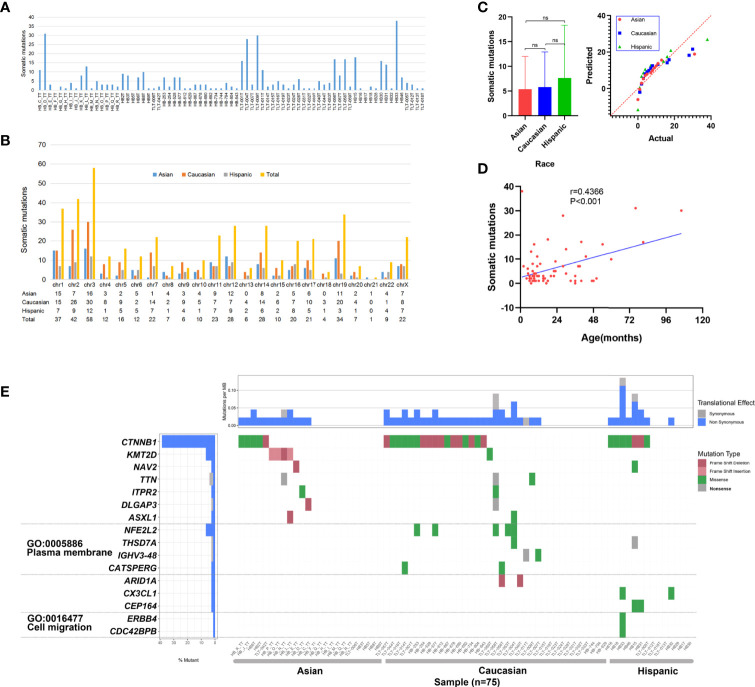
Somatic mutation data of 75 hepatoblastoma (HB) patients. **(A)** Histogram of somatic mutation data for patient of three different races. **(B)** Histogram comparison of somatic mutation number data for each chromosome. **(C)** Statistical analysis of somatic mutation numbers among patients of three races (ns means Asian vs Caucasian: P = 0.841; Asian vs Hispanic: P = 0.387; Caucasian vs Hispanic: P = 0.447; Sidak’s multiple comparisons test). **(D)** The age of disease onset of hepatoblastoma was plotted against the number of somatic mutations, and linear correlation analysis was performed, with the linear regression depicted in blue (r = 0.4366,P < 0.001). **(E)** Somatic mutations in HB patients of three races. The heatmap displays the landscape of genes with recurrent mutations in HB. The central heatmap shows the mutation status of the genes with recurrent mutations for each tumor. Somatic mutations are colored according to functional class (right). Left, mutation count for each individual gene.

To capture the genetic alterations and characterize underlying HB, we performed WES of HB tumors and corresponding noncancerous liver tissues from 16 Chinese patients from our center (**Supplementary Table 1** and **Supplementary Figure 1**). To the best of our knowledge, this is the single largest Chinese population studied in this context, and it is the first time that both preoperative CG and NCG sequencing data have been included. The average sequencing depth of the target region was approximately 167.23× (**Supplementary Tables 2**, **3** and **Supplementary Figure 2**). A total of 92 somatic mutations in 88 genes were detected, with an average of 5.7 mutations per patient. The substitution analysis showed that G>A and C>T substitutions were the most common changes, with 26 and 13 substitutions, respectively (**Figure 2** and **Supplementary Table 4**). For our Chinese samples, *CTNNB1* and *KMT2D* had recurrent mutations, with two or more mutations each and mutation rates of 12.5% and 25%, respectively. The primer sequences of Sanger sequencing are shown in **Supplementary Table 5**. We collected WES data of 65 patients from published studies combined with 16 HB patients from our center, a total of 81 patients with HB were analyzed. After excluding the data of 4 patients with no clear race and of those who were the only patient in their ethnic group, the final number of patients included in our study was 75, including 24 Asian, 37 Caucasian and 14 Hispanic patients. The clinical background of this cohort is shown in **Supplementary Table 6**. The data from multiple ethnic groups showed that a total of 451 somatic mutations in 396 genes were detected in 75 HB patients. The detailed data of the WES set of all studies are shown in **Supplementary Tables 4** and **7**. An average of 6 mutations per patient were observed (**Figure 3A**), indicating that the mutation rate of HB in children is much lower than that of hepatocellular carcinoma in adults (21). Somatic mutations in chromosomes 3, 2, 1 and 19 were the most common (**Figure 3B**). In addition, we confirmed that there was no significant difference in the number of mutations between races (**Figure 3C**), but there was a positive correlation between age and somatic mutation number (r = 0.4366, *P* < 0.001; linear correlation analysis; **Figure 3D**).

### CNA Analysis of 16 Asian HB Patients in Our Center

To study additional gene mutations in HB, we performed a relatively comprehensive genomic copy number spectrum analysisin 16 HB tumors (from our center) and their noncancerous liver tissues. All 16 cases had CNAs. The detailed description is in **Supplementary Table 8**. We identified 1408 CNAs in 16 genomes, including 450 amplifications and 958 deletions. The length of the 1408 CNAs ranged from 42 bp to 149352 bp (median, 10815 bp). The loss of copy number in each HB genome was higher than the gain (2.1:1), with an average of 88 CNAs per HB genome. These data indicate that the incidence of CNA is much higher than that of SNV in tumors in our center.

### Identification of Genes With Recurrent Somatic Mutations and Analysis of GO Enrichment Function in Three Ethnic Groups

Multicenter data (75 patients) analysis showed that 16 genes had recurrent mutations; the *CTNNB1* mutation rate was the highest (28/75; 37.33%), followed by *KMT2D* (5/75; 6.67%) and *NFE2L2* (4/75; 5.33%) (**Figure 3E**). We observed that the mutation rates of *CTNNB1* in the three ancestral groups of Asian, Caucasian and Hispanic populations were different, at 21%, 46% and 50%, respectively. Fisher’s exact test revealed that the mutation rate of Asian patients was significantly lower than that of Caucasian patients (*P* = 0.046). In addition, we observed that there was a high mutation rate in *KMT2D* in Asian data (16.67%), and there were only a few mutations in Caucasians (2.78%). The statistical results showed that there was a significant difference between Asian and Caucasian patients (*P* = 0.05, Fisher’s exact test), while no somatic mutation was detected in Hispanic patients. *NFE2L2* showed mutations only in the Caucasian population (11%), but no somatic mutations were found in the Asian or Hispanic populations. See **Supplementary Table 9** for detailed data analysis.

To detect whether the genes with recurrent mutations are significantly enriched in specific GO terms, we also conducted GO enrichment analysis. The results showed that six genes were related to two GO terms in the cellular component category: the plasma membrane (GO: 0005886) and cell migration (GO: 0016477) (**Figure 3E**). We observed a high mutation rate (24.32%) of plasma membrane-related genes in Caucasians, with a low mutation rate in the Hispanic population (7.14%) and no alterations in the Asian population. The statistical results show that the cell migration-related gene mutation rate in the Asian population is significantly lower than that in the Caucasian (*P* = 0.009, Fisher’s exact test) and Hispanic (*P* = 0.019, Fisher’s exact test) populations. Cell migration term mutations occurred only in one Hispanic patient. See **Supplementary Table 10** for detailed data analysis.

### Significant Oncogenic Pathways in HB

**Figure 4:**
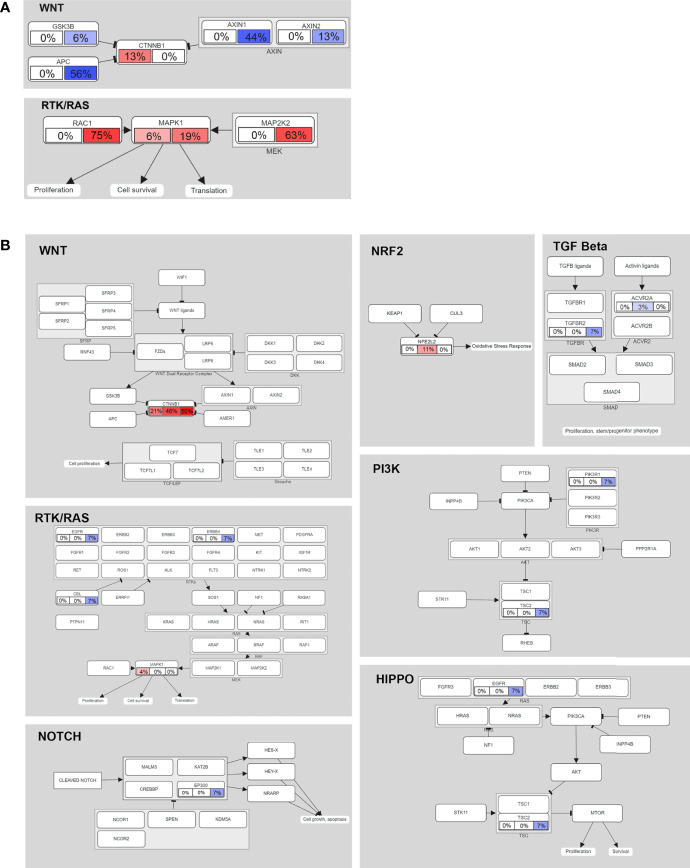
Mutated genes (tumor suppressors indicated in blue, oncogenes indicated in red) and their relationships to other genes, families, complexes, and cellular processes are displayed using curated pathway templates derived from TCGA data. **(A)** Oncogenic network in HB (16 patients in our center). Percentages of somatic mutations and CNAs shown in the left and right portions of each rectangle. **(B)** The oncogenic network of somatic mutations in 75 cases of HB. For each gene, the fraction of mutated samples within the Asian, Caucasian and Hispanic populations is shown.

To further explore the oncogene pathway, we assessed the pathways involving the altered genes. In the data from our center (16 cases), the mutated genes were mainly enriched in the Wnt/β-catenin and receptor tyrosine kinase (RTK)/RAS pathways. *CTNNB1* is still the most important recurrent mutation gene in the Wnt/β-catenin pathway, and the CNA of *APC* and *AXIN1* in the pathway is as high as 56% and 44%, respectively (**Figure 4A**). We also observed CNA in *MAP2K1* and *MAP2K2* of the RTK/RAS pathway at a frequency of 19% and 63%, respectively. In addition, the mutation frequency of *RAC1* was as high as 75% (**Figure 4A**).

In the multiethnic cohort (75 patients), we found that somatic mutant genes were mainly enriched in the following seven pathways: Wnt/β-catenin (38.67%), NRF2 (5.33%), RTK/RAS (4%), HIPPO/β-catenin (2.67%), TGF beta (2.67%), PI3K/Akt (1.33%) and NOTCH (1.33%) (**Figure 4B**). See **Supplementary Table 11** for detailed data analysis.

### Analysis of the Mutational Landscape of the Preoperative CG and NCG of HB Patients

**Figure 5:**
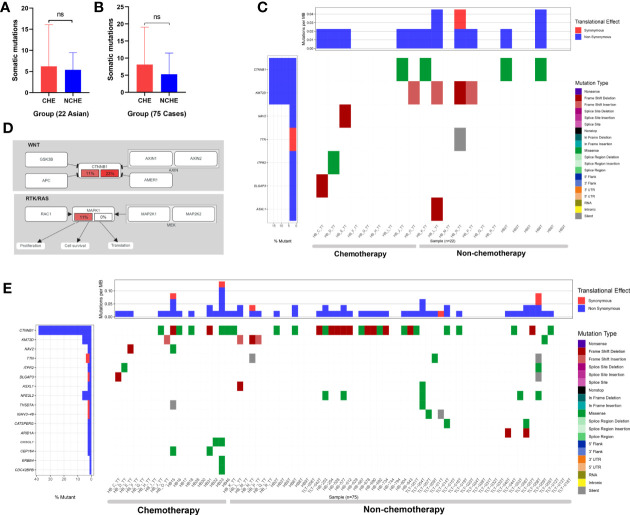
Somatic mutations of HB in the nonchemotherapy group (NCG) and chemotherapy group (CG) before operation. **(A)** Comparative analysis of the somatic mutation number of 22 cases (from Asian) of hepatoblastoma samples from the NCG and CG before surgery (ns means P = 0.785, Mann-Whitney U test). **(B)** Comparative analysis of somatic mutation numbers of 75 cases of hepatoblastoma samples from the NCG and CG before operation (ns means *P* = 0.17, Mann-Whitney U test). **(C)** Somatic mutations of 22 hepatoblastoma patients in the NCG (13 patients) and CG (9 patients) before surgery. The heatmap displays the landscape of recurrently mutated genes in hepatoblastoma. The central heatmap shows the mutation status of the recurrently mutated genes for each tumor. Somatic mutations are colored according to functional class (right). Left, mutation count for each individual gene. **(D)** Oncogenic network in hepatoblastoma (22 Asian patients). The fraction of mutated samples within the preoperative NCG and CG is shown in the left and right portions of each rectangle. **(E)** Somatic mutations of 75 hepatoblastoma patients in the NCG (56 patients) and CG (19 patients) before surgery. The heatmap displays the landscape of recurrently mutated genes in HB. The central heatmap shows the mutation status of the genes with recurrent mutations for each tumor. Somatic mutations are colored according to functional class (right). Left, mutation count for each individual gene.

To evaluate the difference in mutation between preoperative CG and NCG patients, we divided the patients into two groups according to their clinical characteristics. To reduce the analysis bias caused by population differences, we first analyzed the sequencing data of 22 Asian patients (9 in the CG and 13 in the NCG) from the Chinese database. The results showed that there was no significant difference in the number of somatic mutations (*P* = 0.785, Mann-Whitney U test, **Figure 5**) between the two groups, and the mutated genes were mainly enriched in Wnt/β-catenin and RTK/RAS signaling pathways (**Figure 5**, **Supplementary Table 12**). The mutation rate of *CTNNB1* was higher in the NCG (23%) than in the CG (11%), while the mutation rate of *MAPK1* in the RTK/RAS pathway was 11% the CG, but no somatic mutation was observed in the NCG (**Figure 5**).

Among the 75 patients from three ethnic groups, 56 were in the NCG (15 Asian, 37 Caucasian, 4 Hispanic patients) and 19 were in the CG (9 Asian and 10 Hispanic patients). The results showed that there was no significant difference in the number of somatic mutations (*P* = 0.17, Mann-Whitney U test, **Figure 5**) or the number of genes with recurrent mutations between the two groups (**Figure 5**).

In summary, there was no significant difference in the type and frequency of gene changes between preoperative CG and NCG patients, and no significant difference was found in the enrichment of signaling pathways related to mutated genes in the 22 Asian HB patients between the two groups.

### Preliminary Functional Screening of Candidate Cancer-Associated Genes in HB

**Figure 6:**
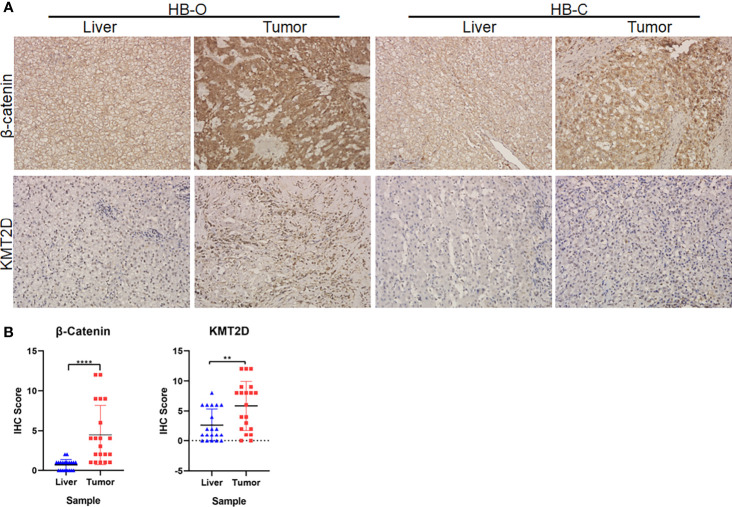
Immunohistochemical (IHC) analyses of β-catenin and KMT2D in HB tumors. **(A)** Representative IHC images of β-catenin and KMT2D staining in two HB tumors and paracancerous normal liver tissues (HB-O and HB-C). Original magnifications, 200×. **(B)** Statistical analysis of the IHC staining of β-catenin and KMT2D proteins in 20 pairs of HB and paracancerous normal liver tissues from our center. The results showed that the expression levels of β-catenin and KMT2D in tumor tissues were significantly higher than those in normal liver tissues (****P < 0.0001, **P = 0.0057, respectively).

In the preliminary validation of the new cancer-associated genes, immunohistochemical staining showed that β-catenin accumulated abnormally in the cytoplasm or nucleus of HB tumors but was rarely expressed in normal liver tissue. There was a significant difference in expression levels between the two tissue types (*P* < 0.0001, Fisher’s exact test, **Figures 6A, B**). In addition, we observed that the protein expression levels of KMT2D in tumors were significantly higher than those in noncancerous liver tissues (*P* = 0.0057, Fisher’s exact test, **Figures 6A, B**).

**Figure 7:**
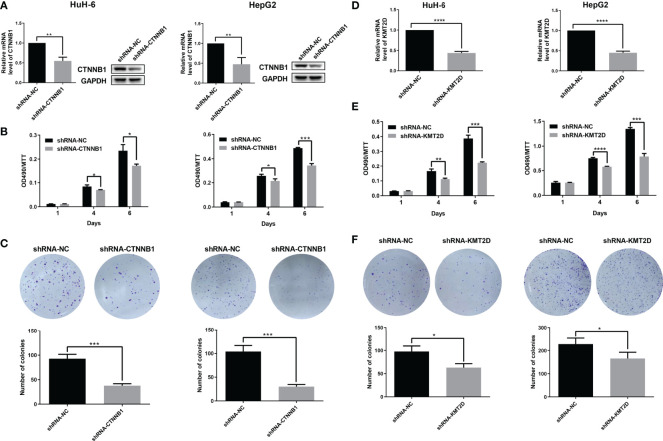
Preliminary functional screening of candidate cancer-associated genes in HB. **(A)** The mRNA and protein levels of *CTNNB1* after lentivirus-mediated knockdown were assessed using qRT-PCR and western blotting in HUH-6 and HepG2 cells. **(B)** Cell proliferation was detected by MTT assays at the indicated days in HUH-6 and HepG2 cells after lentivirus-mediated knockdown of *CTNNB1*. **(C)** Colony formation assay showing the effects of the *CTNNB1* gene knockdown on the proliferation of HUH-6 and HepG2 cells. Experiments were repeated at least three times, each bar shows the mean ± SD (*P < 0.05, **P < 0.01, ***P < 0.001, ****P < 0.0001). **(D)** The mRNA levels of *KMT2D* after lentivirus-mediated knockdown were assessed using qRT-PCR in HUH-6 and HepG2 cells. **(E)** Cell proliferation was detected by MTT assays at the indicated days in HUH-6 and HepG2 cells after lentivirus-mediated knockdown of *KMT2D*. **(F)** Colony formation assay showing the effects of the *KMT2D* gene knockdown on the proliferation of HUH-6 and HepG2 cells. Experiments were repeated at least three times, each bar shows the mean ± SD (*P < 0.05, **P < 0.01, ***P < 0.001, ****P < 0.0001).

In order to further determine the function of two candidate genes *CTNNB1* and *KMT2D*, and locate potential cancer-associated genes that may play a key role in cell proliferation, we used an short hairpin RNA (shRNA)-mediated loss-of-function screening method. We knocked down the *CTNNB1* and *KMT2D* in two HB cell lines, HepG2 and HUH-6. The results showed that which significantly inhibited the proliferation of HepG2 and HUH-6 cells by MTT assays and reduced the colony formation ability (**Figure 7**). Therefore, our study further confirmed the role of *CTNNB1* in HB and suggested that *KMT2D* may be a novel candidate oncogene.

## Discussion

The current trans-ancestry HB genomic study identified, for the first time, the characteristics of gene variations, which may be more prominent among ethnic groups but are not significantly altered between the preoperative CG and NCG patients. These correlations may highlight the deeper genetic diversity and/or environmental factors in HB (14). Except for 10 samples from Brazil, the sequencing depth of other samples was also approximately 150 ×. So we think it doesn’t substantially affect the results because of the different sequencing depths. This study found that there was a positive correlation between the number of somatic mutations and age, which was reflective of the fact that the risk of HB increases with age, especially in patients older than 8 years old, and the prognosis worsens with age (23). This also suggests that gene mutation number may be one of the important factors for prognosis judgment at the biological research level. Although the reasons for these mutation characteristics have not been fully elucidated in HB (24), on the basis of next-generation sequencing technology, we summarized the existence of previously unexplored mutation processes, which may have a positive impact on the understanding of mutations among different ethnic groups and on further trans-ancestry studies (25, 26).

In this study, we first analyzed the WES data of 16 cases of HB from our center and comprehensively analyzed the data of published studies, including three ethnic groups: Asian, Caucasian and Hispanic. A total of 75 cases were included in the study. To the best of our knowledge, this is by far the largest genomic analysis on the WES of HB in children. It enables people to understand HB mutations more comprehensively than ever before. In addition to the identification of a large number of somatic mutation genes, we found that 16 genes were recurrent mutations. *CTNNB1*, an important oncogene in HB (27), had the highest mutation rate in this study. It has been reported that the mutation rate of *CTNNB1* is 13-87% in HB, and the deletion rate is 0-53% (28–30). Among the 75 patients in this study, the mutation rate was 37.33%. The highest mutation rate was found in the Hispanic population (50%), which was twice that in the Asian population (21%). In addition, we found that *KMT2D* was the second most frequently mutated gene, and the abnormally high expression of KMT2D protein was confirmed by further immunohistochemical analysis. This gene has been reported in many kinds of tumors, such as gastric cancer and bladder cancer, and it is believed that this gene is closely related to the pathogenesis of tumors (31, 32). However, Chavan et al. (33) reported that no *KMT2D* mutation was detected in 18 HB samples from their centers. We believe that this may be due to the limited sample size of a single center because we collected data from multiple centers and large samples to reduce this bias.

In the analysis of GO function, we found that the mutation rate of plasma membrane (GO: 0005886) term-related genes in the Caucasian and Hispanic populations was significantly higher than that in the Asian population. As a platform of intracellular and intercellular redox signals, the plasma membrane plays a key role in tumorigenesis and development (34, 35). We found that the Wnt/β-catenin pathway, a well-known pathway in tumorigenesis (36–38), was the most frequently mutated pathway (10). However, the frequency of mutations in Caucasians and Hispanics was approximately twice as high as that in Asians. The NRF2 pathway was also found to have a high frequency of alteration (5.33%); this pathway includes *NFE2L2*, a key transcription regulator of the oxidative stress response (39, 40), which was mutated in four Caucasians (11%). Some studies have suggested that this gene and *CTNNB1* are often mutated simultaneously in HB (41), but in our study, only in one patient (HB-253) had both mutations at the same time. In any case, it should be noted that this gene has been confirmed to be closely related to the prognosis of HB (41, 42), which indicates that this pathway may plays an important role in HB. In addition, the mutation rates of the RTK/RAS and HIPPO/β-catenin pathway-related genes were the highest in Hispanic patients. Mutations in the RTK/Ras pathway are found in 50% of tumors, and the frequency of mutations is different among different tumors (43). This pathway is often a key dysregulated pathway in lung adenocarcinoma, and many of its members have become targets for small molecule kinase inhibitor therapy (43–46). In this study, we observed that the mutation frequency of the RTK/Ras pathway differed among HB patients of different races. The HIPPO/β-catenin pathways regulate the size, proliferation and survival of normal organs and tissues and affects tumor growth (47). Mutations in the HIPPO/β-catenin pathway have been detected in 14% of Hispanic patients. Yes-associated protein (YAP) is a part of the HIPPO/β-catenin tumor inhibition pathway (48, 49). It has been confirmed that HIPPO/β-catenin pathway inactivation causes nuclear translocation of YAP, which interacts with β-catenin in HB (50, 51). These results may have a certain guiding role in the development of targeted drugs and their application in different populations of patients.

We verified the function of some candidate genes and tried to find new cancer-associated genes. In the gene verification of somatic mutation, *CTNNB1* knockdown significantly inhibited the growth of HB cells, which confirmed its important role in HB. In addition, it is exciting to find a new candidate gene *KMT2D*. Moreover, the results of cell function test show that *KMT2D* knockdown can significantly inhibit the growth of HB cells, which further suggests that which may be a novel candidate oncogene in HB.

Despite the comprehensive efforts of this study, there are still a few limitations. First, Caucasians account for a large proportion of the various ethnic groups in the study, which may cause some bias in the analysis results. Second, because we could not obtain the tumor samples of patients who underwent preoperative chemotherapy at the time of the initial diagnosis, we could not determine whether the gene mutation detected after chemotherapy was a preexisting mutation or a new mutation. However, after we combined the multicenter CG and NCG samples, this influence was reduced, so it did not affect our study of gene mutations in patients after chemotherapy. Third, the functions of the new candidate cancer-associated genes need to be verified with further *in vivo* and *in vitro* experiments on *KMT2D*.

## Conclusion

In conclusion, we found that there were ethnic differences in somatic mutations among Asian, Caucasian and Hispanic patients with HB, but there was no significant difference in gene mutations before and after chemotherapy. The results indicate that there are ethnic differences in HB biology at the genomic level, which expands our understanding of the genetics of HB in children. In addition, *KMT2D* may be a novel candidate oncogene for HB.

## Data Availability Statement

The datasets presented in this study can be found in online repositories. The names of the repository/repositories and accession number(s) can be found in the article/**Supplementary Material**.

## Ethics Statement

The studies involving human participants were reviewed and approved by the Ethics Committee of the Affiliated Hospital of Qingdao University. Written informed consent to participate in this study was provided by the participants’ legal guardian/next of kin.

## Author Contributions

All authors contributed to the article and approved the submitted version. QD, ZL and JL designed the research. JL, CG, LW, XJ, and QZ performed the experiments. XH, YC, MM, TL, and HN obtained the clinical data. JieZ and NX made immunohistochemical staining and analysis of the results. JL, CG and LW prepared the tables and figures. JL, CG, and LW prepared the manuscript. ZL and QD revised the manuscript.

## Funding

This study was funded by the National Science and Technology Major Project of the Ministry of Science and Technology of China (No. 2020ZX09201-018), the Qingdao Civic Science and Technology Program (No. 17-3-3-8-nsh). The funders had no role in the design of the study and collection, analysis, and interpretation of data and in writing the manuscript.

## Conflict of Interest

The authors declare that the research was conducted in the absence of any commercial or financial relationships that could be construed as a potential conflict of interest.

## Abbreviations

HB, hepatoblastoma; WES, whole-exome sequencing; FFPE, Formalin-fixed paraffin-embedded; CNA, Copy number alteration; SNV, single nucleotide variant; GO, Gene Oncology; KEGG, Kyoto Encyclopedia of Genes and Genomes; CG, chemotherapy group; IHC, immunohistochemical.
