# Supplementary material for: Trans-Ancestry Mutation Landscape of Hepatoblastoma Genomes in Children
Source: Front Oncol. 2021 Apr 21;11:669560. doi: 10.3389/fonc.2021.669560 (PMC8096978; doi:10.3389/fonc.2021.669560)
Supplement: Supplementary file 1 [file DataSheet_1.doc]

**
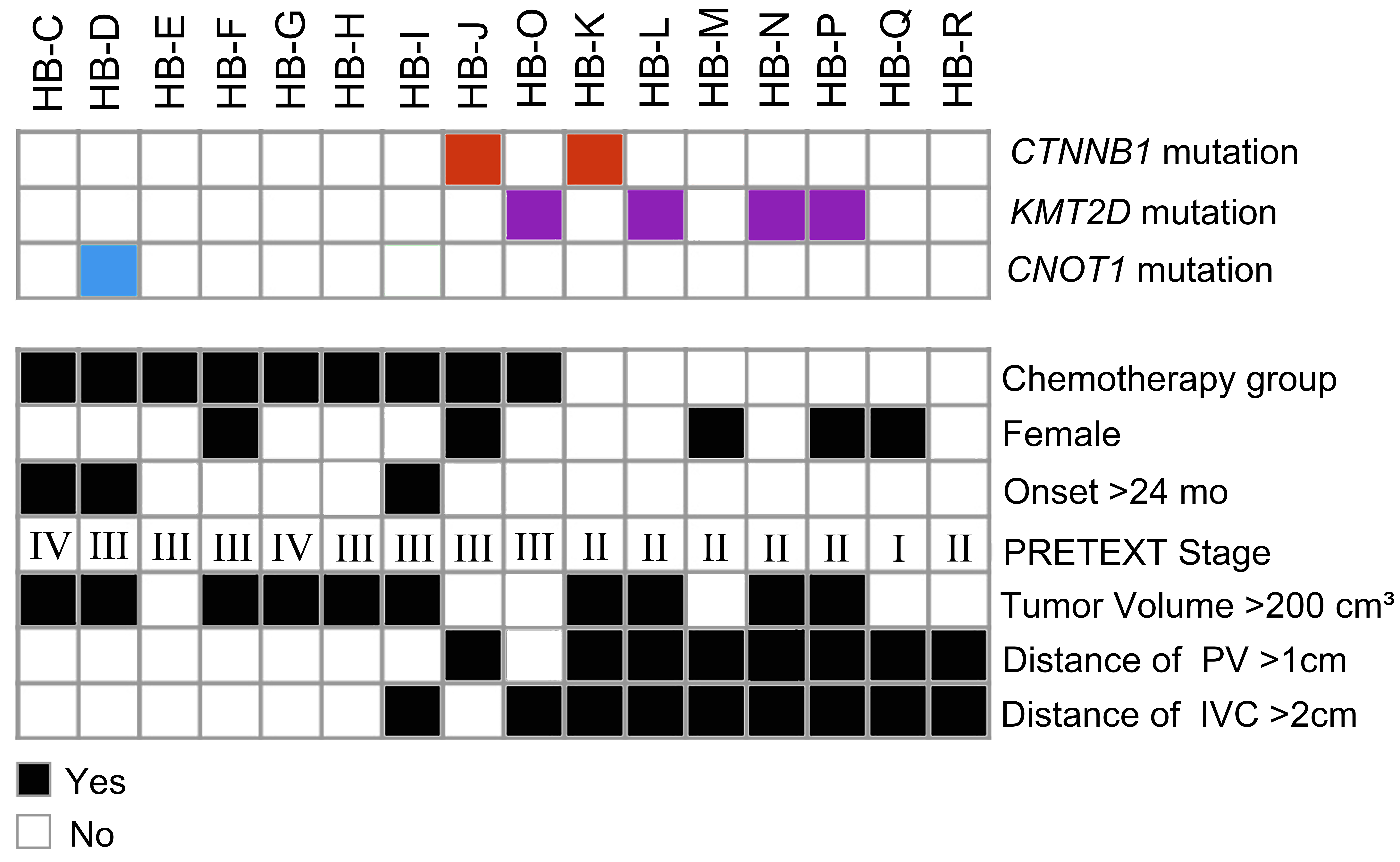
**

**Supplementary Fig. 1** Clinic characteristics and the mutational status of the *CTNNB1*, *KMT2D*, and *CNOT1* genes are color-coded and depicted in rows for each tumour of our cohort of 16 hepatoblastoma (HB) patients.

**
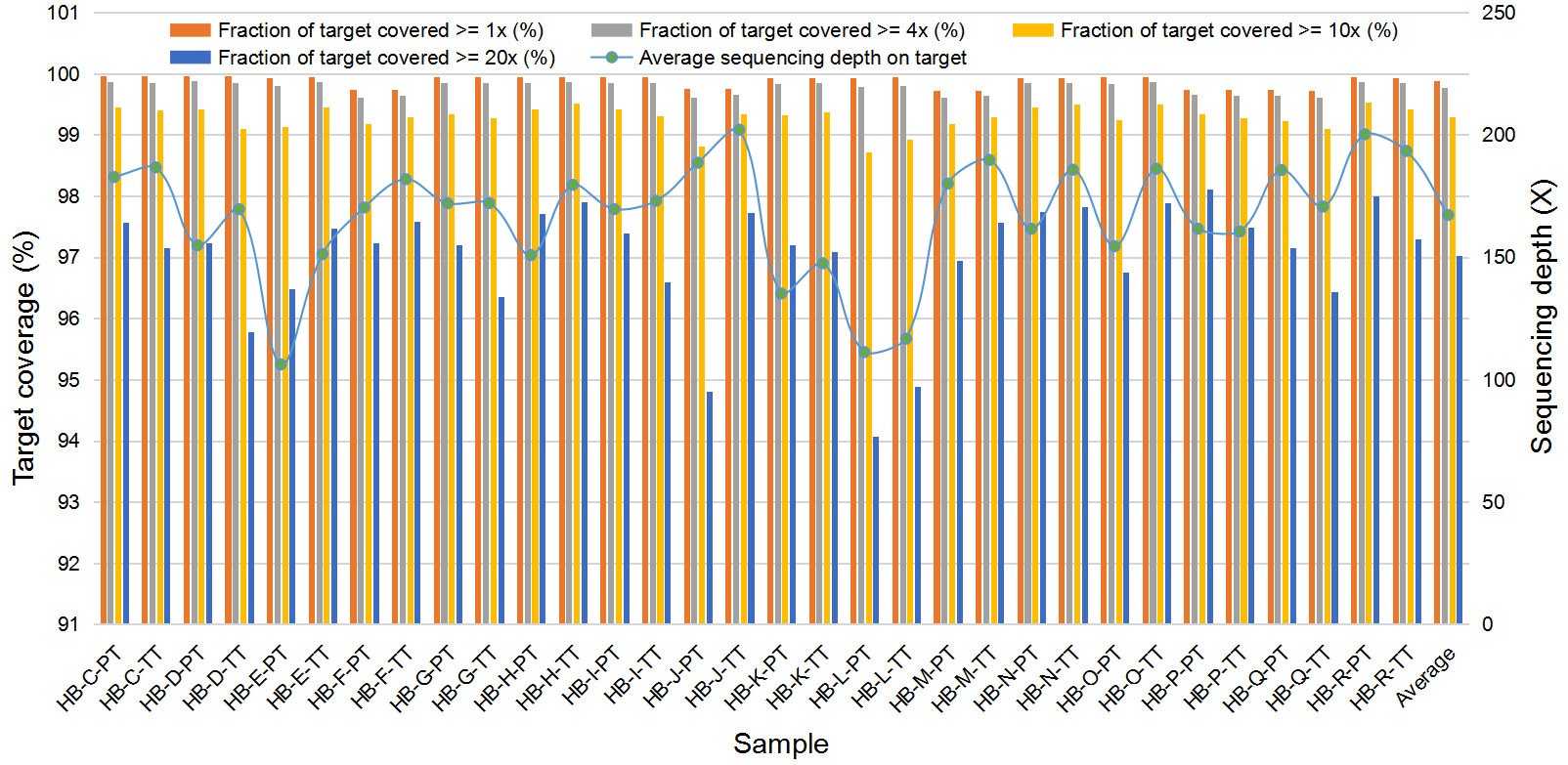
**

**Supplementary Fig. 2** Summary of the distribution of per-base sequencing depth on targets of 16 HB patients at our center.
