# Supplementary material for: Trans-Ancestry Mutation Landscape of Hepatoblastoma Genomes in Children
Source: Front Oncol. 2021 Apr 21;11:669560. doi: 10.3389/fonc.2021.669560 (PMC8096978; doi:10.3389/fonc.2021.669560)
Supplement: Supplementary file 2 [file Table_1.doc]

Supplementary Table 1 Summary of Clinical Characteristics for the Set of 16 hepatoblastoma Patients in our center

| Case No. | Gender | Age  (mo) | AFP (ng/ml) | Location of  tumor | Histology Type | pre-operative chemotherapy cycle |
| --- | --- | --- | --- | --- | --- | --- |
| HB-C | M | 43 | 686400 | Right lobe | Epithelial，fetal | 3 |
| HB-D | M | 76 | 782230 | Right lobe | Epithelial;embryonal | 4 |
| HB-E | M | 8 | 282250 | Right lobe | Epithelial;fetal | 3 |
| HB-F | F | 17 | 420190 | Right lobe | Epithelial; fetal, embryonal | 2 |
| HB-G | M | 16 | 10223 | Middle lobe | Mixed ,epithelial and mesenchymal | 3 |
| HB-H | M | 18 | 319274 | Right lobe | Mixed;epithelial and mesenchymal | 3 |
| HB-I | M | 30 | 742020 | Three right lobe | Epithelial;embryonal | 3 |
| HB-J | F | 8 | 330509 | Three right lobe | Mixed ;epithelial and mesenchymal | 4 |
| HB-O | M | 22 | 296250 | Middle lobe | Epithelial; fetal, embryonal | 2 |
| HB-K | M | 8 | >1210 | Three right lobe | Epithelial;fetal |  |
| HB-L | M | 7 | 72969 | Right lobe | Epithelial;embryonal |  |
| HB-M | F | 9 | 5173 | Right lobe | Epithelial;fetal |  |
| HB-N | M | 23 | >1210 | Right lobe | Epithelial;embryonal |  |
| HB-P | F | 15 | 123012 | Right lobe | Epithelial, embryonic and fetal types are all visible |  |
| HB-Q | F | 12 | 5426 | V,VI | Epithelial;fetal |  |
| HB-R | M | 7 | 20238 | V,VI | Mixed;epithelial and mesenchymal |  |

Abbreviations: non-chemotherapy group HB Patients: HB-K, HB-L, HB-M, HB-N, HB-P, HB-Q, HB-R.

chemotherapy group HB Patients: HB-C, HB-D, HB-E, HB-F, HB-G, HB-H, HB-I, HB-J, HB-O.

| Supplementary Table 2 Summary of the distribution of per-base sequencing depth on targets of 16 HB patients at our center. | | | | | | | | | | |
| --- | --- | --- | --- | --- | --- | --- | --- | --- | --- | --- |
| Samples | Raw reads | Raw bases (Mb) | Clean reads | Clean bases (Mb) | Clean data rate (%) | Clean read1 Q20 (%) | Clean read2 Q20 (%) | Clean read1 Q30 (%) | Clean read2 Q30 (%) | GC content (%) |
| HB_C_PT | 263554900 | 26355.49 | 263488478 | 26258.91 | 99.63 | 97.2 | 95.46 | 89.85 | 86.55 | 56.11 |
| HB_C_TT | 269341120 | 26934.11 | 269280154 | 26857.02 | 99.71 | 97.3 | 96.07 | 90.17 | 87.93 | 55.95 |
| HB_D_PT | 252023776 | 25202.38 | 251974768 | 25135.9 | 99.74 | 97.88 | 96.84 | 91.08 | 89.05 | 55.3 |
| HB_D_TT | 261341922 | 26134.19 | 261293004 | 26056.08 | 99.7 | 97.89 | 96.84 | 91.11 | 89.01 | 56.93 |
| HB_E_PT | 233780608 | 23378.06 | 233247756 | 23163.09 | 99.08 | 98.1 | 97.68 | 92.02 | 91.55 | 53.22 |
| HB_E_TT | 259743848 | 25974.38 | 259695646 | 25914.82 | 99.77 | 97.96 | 97.11 | 91.42 | 89.84 | 55.3 |
| HB_F_PT | 262652424 | 26265.24 | 262604060 | 26201.15 | 99.76 | 97.91 | 96.77 | 91.11 | 88.75 | 55.77 |
| HB_F_TT | 275193194 | 27519.32 | 275149732 | 27472.05 | 99.83 | 97.99 | 97.37 | 91.42 | 90.43 | 54.19 |
| HB_G_PT | 261668978 | 26166.9 | 261613776 | 26025.2 | 99.46 | 97.95 | 96.94 | 91.42 | 89.43 | 56.66 |
| HB_G_TT | 268221766 | 26822.18 | 268175710 | 26755.05 | 99.75 | 97.91 | 97.05 | 91.14 | 89.47 | 56.12 |
| HB_H_PT | 236080290 | 23608.03 | 236033646 | 23520.5 | 99.63 | 97.96 | 97.11 | 91.35 | 89.78 | 53.6 |
| HB_H_TT | 260576438 | 26057.64 | 260527162 | 26003.44 | 99.79 | 97.84 | 97.02 | 90.98 | 89.46 | 55.39 |
| HB_I_PT | 259169942 | 25916.99 | 259111556 | 25818.1 | 99.62 | 97.99 | 97.3 | 91.55 | 90.43 | 55.99 |
| HB_I_TT | 264505832 | 26450.58 | 264454850 | 26400.76 | 99.81 | 97.85 | 96.81 | 90.94 | 88.96 | 55.99 |
| HB_J_PT | 257007786 | 25700.78 | 256958600 | 25629.95 | 99.72 | 97.86 | 96.91 | 91.03 | 89.16 | 57.2 |
| HB_J_TT | 270827960 | 27082.8 | 270778056 | 27044.92 | 99.86 | 97.82 | 96.79 | 90.8 | 88.8 | 55.25 |
| HB_K_PT | 255335988 | 25533.6 | 255289306 | 25490.27 | 99.83 | 98.05 | 97.51 | 91.78 | 90.93 | 52.07 |
| HB_K_TT | 250224594 | 25022.46 | 250178922 | 24943.34 | 99.68 | 97.98 | 97.45 | 91.6 | 90.89 | 54.97 |
| HB_L_PT | 256793098 | 25679.31 | 256724660 | 25514.23 | 99.36 | 98.04 | 97.27 | 91.81 | 90.3 | 54.54 |
| HB_L_TT | 262538200 | 26253.82 | 262476802 | 26159.34 | 99.64 | 97.98 | 96.88 | 91.51 | 89.23 | 55.03 |
| HB_M_PT | 253607698 | 25360.77 | 253559938 | 25302.91 | 99.77 | 97.75 | 96.95 | 90.81 | 89.32 | 56.52 |
| HB_M_TT | 259420894 | 25942.09 | 259370088 | 25881.77 | 99.77 | 97.9 | 97.03 | 91.25 | 89.65 | 56.29 |
| HB_N_PT | 250420746 | 25042.07 | 250369072 | 24985.64 | 99.77 | 97.99 | 97.15 | 91.5 | 89.95 | 54.31 |
| HB_N_TT | 256202290 | 25620.23 | 256153194 | 25553.56 | 99.74 | 97.89 | 96.97 | 91.17 | 89.41 | 55.39 |
| HB_O_PT | 215498344 | 21549.83 | 215459536 | 21415.47 | 99.38 | 98.1 | 97.26 | 92 | 90.34 | 55.89 |
| HB_O_TT | 251165292 | 25116.53 | 251117888 | 24981.01 | 99.46 | 98.05 | 97.2 | 91.79 | 90.13 | 55.19 |
| HB_P_PT | 261140516 | 26114.05 | 261049446 | 26057.68 | 99.78 | 97.16 | 95.58 | 89.63 | 86.84 | 53.95 |
| HB_P_TT | 271569478 | 27156.95 | 271469458 | 27093.65 | 99.77 | 97.2 | 96.03 | 89.92 | 87.95 | 55.8 |
| HB_Q_PT | 267163056 | 26716.31 | 267092940 | 26670 | 99.83 | 96.98 | 95.34 | 89.28 | 86.36 | 56.75 |
| HB_Q_TT | 263444602 | 26344.46 | 263354400 | 26300.58 | 99.83 | 97.15 | 95.67 | 89.66 | 87.01 | 57.12 |
| HB_R_PT | 268214860 | 26821.49 | 268149898 | 26774.35 | 99.82 | 97.18 | 96.31 | 89.84 | 88.54 | 55.41 |
| HB_R_TT | 266047916 | 26604.79 | 265993902 | 26568.35 | 99.86 | 97.34 | 96.15 | 90.15 | 88.03 | 55.95 |
| Average | 258264948 | 25826.49 | 258193637 | 25748.41 | 99.69 | 97.75 | 96.78 | 90.97 | 89.17 | 55.44 |

| Supplementary Table 3 Whole-exome sequencing data quality control for 16 HB patients samples in our center. |
| --- |

| Samples | Initial bases on target | Total effective reads | Total effective bases (Mb) | Effective sequences on target (Mb) | Capture specificity (%) | Mapping rate on genome (%) | Duplicate rate on genome (%) | Mismatch rate in target region (%) | Average sequencing depth on target | Fraction of target covered >= 1x (%) | Fraction of target covered >= 4x (%) | Fraction of target covered >= 10x (%) | Fraction of target covered >= 20x (%) |
| --- | --- | --- | --- | --- | --- | --- | --- | --- | --- | --- | --- | --- | --- |
| HB_C_PT | 60456963 | 150497183 | 14570.3 | 11047.7 | 75.82 | 99.82 | 43.21 | 0.49 | 182.74 | 99.96 | 99.87 | 99.45 | 97.57 |
| HB_C_TT | 60456963 | 163388708 | 15821.1 | 11286 | 71.34 | 99.84 | 39.76 | 0.46 | 186.68 | 99.96 | 99.86 | 99.4 | 97.15 |
| HB_D_PT | 60456963 | 133965766 | 12932.9 | 9362.14 | 72.39 | 99.93 | 47.32 | 0.31 | 154.86 | 99.97 | 99.89 | 99.42 | 97.24 |
| HB_D_TT | 60456963 | 140066324 | 13573.8 | 10253 | 75.54 | 99.93 | 46.79 | 0.31 | 169.59 | 99.96 | 99.85 | 99.1 | 95.78 |
| HB_E_PT | 60456963 | 99903443 | 9141.11 | 6418.68 | 70.22 | 99.9 | 58 | 0.31 | 106.17 | 99.94 | 99.81 | 99.14 | 96.49 |
| HB_E_TT | 60456963 | 129729541 | 12530.4 | 9142.56 | 72.96 | 99.92 | 50.47 | 0.31 | 151.22 | 99.95 | 99.87 | 99.45 | 97.47 |
| HB_F_PT | 60456963 | 142636655 | 13873.2 | 10296.7 | 74.22 | 99.92 | 46.03 | 0.3 | 170.31 | 99.74 | 99.62 | 99.19 | 97.23 |
| HB_F_TT | 60456963 | 166013451 | 16316.4 | 10993.8 | 67.38 | 99.93 | 39.88 | 0.29 | 181.85 | 99.74 | 99.64 | 99.3 | 97.59 |
| HB_G_PT | 60456963 | 139203986 | 13424.8 | 10400.2 | 77.47 | 99.93 | 47.15 | 0.3 | 172.03 | 99.95 | 99.85 | 99.34 | 97.2 |
| HB_G_TT | 60456963 | 154791884 | 15104.3 | 10405.2 | 68.89 | 99.94 | 42.59 | 0.31 | 172.11 | 99.95 | 99.85 | 99.28 | 96.36 |
| HB_H_PT | 60456963 | 135142455 | 12955.9 | 9119.11 | 70.39 | 99.92 | 43.39 | 0.3 | 150.84 | 99.95 | 99.85 | 99.43 | 97.71 |
| HB_H_TT | 60456963 | 153076556 | 14900.3 | 10854.2 | 72.85 | 99.93 | 41.66 | 0.3 | 179.54 | 99.95 | 99.87 | 99.52 | 97.91 |
| HB_I_PT | 60456963 | 148795495 | 14372.8 | 10256.2 | 71.36 | 99.93 | 43.05 | 0.3 | 169.64 | 99.95 | 99.86 | 99.42 | 97.4 |
| HB_I_TT | 60456963 | 153691651 | 14923.8 | 10460.1 | 70.09 | 99.92 | 42.34 | 0.31 | 173.02 | 99.95 | 99.86 | 99.31 | 96.59 |
| HB_J_PT | 60456963 | 156721217 | 15354.8 | 11400.4 | 74.25 | 99.92 | 39.26 | 0.32 | 188.57 | 99.75 | 99.61 | 98.81 | 94.8 |
| HB_J_TT | 60456963 | 167981354 | 16561.7 | 12215.8 | 73.76 | 99.93 | 38.15 | 0.32 | 202.06 | 99.75 | 99.66 | 99.34 | 97.73 |
| HB_K_PT | 60456963 | 138610148 | 13372.3 | 8168.97 | 61.09 | 99.88 | 46.21 | 0.32 | 135.12 | 99.94 | 99.84 | 99.32 | 97.2 |
| HB_K_TT | 60456963 | 132701225 | 12677.9 | 8917.34 | 70.34 | 99.92 | 47.59 | 0.31 | 147.5 | 99.94 | 99.85 | 99.37 | 97.09 |
| HB_L_PT | 60456963 | 124755905 | 11516.4 | 6723.69 | 58.38 | 99.8 | 52.23 | 0.33 | 111.21 | 99.94 | 99.79 | 98.72 | 94.07 |
| HB_L_TT | 60456963 | 120281823 | 11217.3 | 7054.8 | 62.89 | 99.88 | 54.91 | 0.32 | 116.69 | 99.95 | 99.81 | 98.92 | 94.89 |
| HB_M_PT | 60456963 | 148638090 | 14497.5 | 10886 | 75.09 | 99.93 | 41.74 | 0.33 | 180.06 | 99.73 | 99.62 | 99.18 | 96.94 |
| HB_M_TT | 60456963 | 156360803 | 15231.2 | 11470.6 | 75.31 | 99.92 | 40.13 | 0.33 | 189.73 | 99.73 | 99.64 | 99.29 | 97.57 |
| HB_N_PT | 60456963 | 142274339 | 13731.7 | 9769.87 | 71.15 | 99.9 | 43.71 | 0.32 | 161.6 | 99.94 | 99.85 | 99.45 | 97.75 |
| HB_N_TT | 60456963 | 158407910 | 15414.5 | 11229.1 | 72.85 | 99.93 | 38.6 | 0.33 | 185.74 | 99.94 | 99.86 | 99.51 | 97.82 |
| HB_O_PT | 60456963 | 132888840 | 12693.2 | 9339.07 | 73.58 | 99.91 | 39.02 | 0.33 | 154.47 | 99.95 | 99.83 | 99.24 | 96.75 |
| HB_O_TT | 60456963 | 160893562 | 15526.7 | 11253 | 72.48 | 99.92 | 36.46 | 0.33 | 186.13 | 99.95 | 99.87 | 99.51 | 97.89 |
| HB_P_PT | 60456963 | 138031007 | 13374.6 | 9771.45 | 73.06 | 99.82 | 47.46 | 0.47 | 161.63 | 99.74 | 99.66 | 99.35 | 98.12 |
| HB_P_TT | 60456963 | 141176789 | 13636.6 | 9703.43 | 71.16 | 99.82 | 48.37 | 0.45 | 160.5 | 99.74 | 99.65 | 99.28 | 97.49 |
| HB_Q_PT | 60456963 | 155900895 | 15125.6 | 11220.3 | 74.18 | 99.8 | 42.03 | 0.53 | 185.59 | 99.74 | 99.64 | 99.23 | 97.15 |
| HB_Q_TT | 60456963 | 141010281 | 13676.6 | 10317.8 | 75.44 | 99.79 | 46.8 | 0.46 | 170.66 | 99.73 | 99.62 | 99.1 | 96.44 |
| HB_R_PT | 60456963 | 161281657 | 15889.3 | 12105.4 | 76.19 | 99.83 | 39.94 | 0.46 | 200.23 | 99.95 | 99.87 | 99.54 | 98 |
| HB_R_TT | 60456963 | 154971201 | 15268.2 | 11692.1 | 76.58 | 99.85 | 41.85 | 0.43 | 193.4 | 99.94 | 99.86 | 99.42 | 97.3 |
| Average | 60456963 | 145118442 | 14037.73 | 10110.46 | 71.83 | 99.89 | 44.25 | 0.35 | 167.23 | 99.88 | 99.78 | 99.29 | 97.02 |

Abbreviations: PT: paracancerous tissues; TT: tumor tissues.

| Supplementary Table 4 | | | | | | | | | | | | | | |
| --- | --- | --- | --- | --- | --- | --- | --- | --- | --- | --- | --- | --- | --- | --- |
| Sample | CHROM | POS | REF | ALT | set | AC | AF | NORMAL.GT | NORMAL.AD | TUMOR.GT | TUMOR.AD | HGVSc | HGVSp | Gene |
| HB_O_TT | chr3 | 53186688 | C | T | Intersection | 1 | 0.25 | C/C | 379,0 | C/T | 543,149 | ENST00000330452.8:c.1345C>T | ENSP00000331602.3:p.Arg449Cys | PRKCD |
| HB_O_TT | chr3 | 130431786 | G | A | Intersection | 1 | 0.25 | G/G | 161,0 | G/A | 101,31 | ENST00000265379.10:c.5558G>A | ENSP00000265379.7:p.Arg1853Gln | COL6A5 |
| HB_O_TT | chr11 | 30012379 | G | A | mutect | 1 | 0.25 | G/G | 179,0 | G/A | 213,17 | ENST00000328224.7:c.300C>T | ENSP00000328511.6:p.Ser100= | KCNA4 |
| HB_O_TT | chr12 | 49026797 | A | ACAGGTC | Intersection | 1 | 0.25 | A/A | 491,0 | A/ACAGGTC | 522,108 | ENST00000301067.11:c.15163_15168dup | ENSP00000301067.7:p.Asp5055_Leu5056dup | KMT2D |
| HB_D_TT | chr1 | 2502121 | G | T | mutect | 1 | 0.25 | G/G | 384,0 | G/T | 267,28 | ENST00000378486.8:c.2671G>T | ENSP00000367747.3:p.Ala891Ser | PLCH2 |
| HB_D_TT | chr1 | 2645320 | G | A | Intersection | 1 | 0.25 | G/G | 384,0 | G/A | 347,36 | ENST00000637179.1:c.931C>T | ENSP00000490537.1:p.Leu311Phe | TTC34 |
| HB_D_TT | chr1 | 23749894 | G | A | Intersection | 1 | 0.25 | G/G | 171,0 | G/A | 103,16 | ENST00000418390.6:c.263G>A | ENSP00000395574.2:p.Gly88Glu | ELOA |
| HB_D_TT | chr1 | 27850185 | G | A | mutect | 1 | 0.25 | G/G | 263,1 | G/A | 229,25 | ENST00000311772.10:c.795G>A | ENSP00000311677.5:p.Gly265= | PPP1R8 |
| HB_D_TT | chr1 | 94041227 | G | A | Intersection | 1 | 0.25 | G/G | 137,0 | G/A | 137,14 | ENST00000370225.4:c.3504C>T | ENSP00000359245.3:p.Ser1168= | ABCA4 |
| HB_D_TT | chr1 | 161629880 | T | A | Intersection | 1 | 0.25 | T/T | 298,0 | T/A | 384,40 | ENST00000531221.7:c.325A>T | ENSP00000433642.1:p.Ser109Cys | FCGR3B |
| HB_D_TT | chr1 | 201115158 | G | A | mutect | 1 | 0.25 | G/G | 521,0 | G/A | 803,54 | ENST00000449188.2:c.215C>T | ENSP00000472681.1:p.Ala72Val | ASCL5 |
| HB_D_TT | chr3 | 143034964 | G | A | Intersection | 1 | 0.25 | G/G | 109,0 | G/A | 38,40 | ENST00000473835.7:c.1930G>A | ENSP00000418563.1:p.Glu644Lys | U2SURP |
| HB_D_TT | chr6 | 100448236 | C | T | mutect | 1 | 0.25 | C/C | 140,0 | C/T | 133,11 | ENST00000369208.7:c.760G>A | ENSP00000358210.3:p.Gly254Arg | SIM1 |
| HB_D_TT | chr8 | 144333996 | G | A | mutect | 1 | 0.25 | G/G | 153,0 | G/A | 151,13 | ENST00000569446.3:c.236C>T | ENSP00000455711.1:p.Pro79Leu | SCRT1 |
| HB_D_TT | chr9 | 35724655 | C | T | Intersection | 1 | 0.25 | C/C | 51,0 | C/T | 51,9 | ENST00000314888.10:c.428G>A | ENSP00000316029.9:p.Gly143Glu | TLN1 |
| HB_D_TT | chr10 | 91251351 | G | T | Intersection | 1 | 0.25 | G/G | 35,0 | G/T | 29,11 | ENST00000336126.6:c.385G>T | ENSP00000337500.5:p.Asp129Tyr | PCGF5 |
| HB_D_TT | chr11 | 5666020 | G | A | mutect | 1 | 0.25 | G/G | 102,0 | G/A | 58,6 | ENST00000380034.8:c.829C>T | ENSP00000369373.3:p.Arg277Ter | TRIM5 |
| HB_D_TT | chr11 | 15200930 | G | T | Intersection | 1 | 0.25 | G/G | 97,0 | G/T | 33,47 | ENST00000379554.7:c.941G>T | ENSP00000368872.3:p.Gly314Val | INSC |
| HB_D_TT | chr12 | 900570 | G | GT | Intersection | 1 | 0.25 | G/G | 202,0 | G/GT | 164,72 | ENST00000340908.8:c.7301dup | ENSP00000341292.5:p.Leu2434PhefsTer5 | WNK1 |
| HB_D_TT | chr12 | 26487097 | T | C | mutect | 1 | 0.25 | T/T | 125,0 | T/C | 116,12 | ENST00000381340.8:c.5525A>G | ENSP00000370744.3:p.Glu1842Gly | ITPR2 |
| HB_D_TT | chr14 | 77931531 | G | A | Intersection | 1 | 0.25 | G/G | 419,2 | G/A | 362,127 | ENST00000238561.10:c.1220G>A | ENSP00000238561.5:p.Arg407His | ADCK1 |
| HB_D_TT | chr14 | 99174341 | G | A | Intersection | 1 | 0.25 | G/G | 761,0 | G/A | 369,331 | ENST00000357195.8:c.2495C>T | ENSP00000349723.3:p.Ala832Val | BCL11B |
| HB_D_TT | chr14 | 106268793 | CC | AT | mutect | 1 | 0.25 | CC/CC | 594,0 | CC/AT | 732,31 | ENST00000390609.3:c.165_166delinsAT | ENSP00000375018.2:p.Trp55_Val56delinsTer | IGHV3-23 |
| HB_D_TT | chr16 | 58575007 | C | G | Intersection | 1 | 0.25 | C/C | 57,0 | C/G | 8,9 | ENST00000317147.10:c.1827G>C | ENSP00000320949.5:p.Gly609= | CNOT1 |
| HB_D_TT | chr16 | 72124881 | C | G | mutect | 1 | 0.25 | C/C | 142,0 | C/G | 147,11 | ENST00000237353.15:c.2475G>C | ENSP00000237353.10:p.Trp825Cys | PMFBP1 |
| HB_D_TT | chr19 | 2476605 | T | C | Intersection | 1 | 0.25 | T/T | 238,2 | T/C | 212,69 | ENST00000215631.9:c.121T>C | ENSP00000215631.3:p.Tyr41His | GADD45B |
| HB_D_TT | chr19 | 3637415 | C | G | Intersection | 1 | 0.25 | C/C | 968,0 | C/G | 965,230 | ENST00000589578.5:c.2119G>C | ENSP00000466363.1:p.Gly707Arg | PIP5K1C |
| HB_D_TT | chr19 | 8905004 | C | A | varscan | 1 | 0.25 | C/C | 204,1 | C/A | 150,105 | ENST00000397910.8:c.38143G>T | ENSP00000381008.2:p.Asp12715Tyr | MUC16 |
| HB_D_TT | chr19 | 9214678 | G | T | varscan | 1 | 0.25 | G/G | 92,0 | G/T | 95,15 | ENST00000641244.1:c.160C>A | ENSP00000493404.1:p.His54Asn | OR7D4 |
| HB_D_TT | chr19 | 12752665 | C | G | mutect | 1 | 0.25 | C/C | 207,0 | C/G | 189,11 | ENST00000549706.5:c.73C>G | ENSP00000448310.1:p.Arg25Gly | BEST2 |
| HB_D_TT | chr19 | 15453478 | G | T | Intersection | 1 | 0.25 | G/G | 151,0 | G/T | 158,18 | ENST00000343625.12:c.2299C>A | ENSP00000341905.5:p.Pro767Thr | RASAL3 |
| HB_D_TT | chr19 | 31277161 | TG | T | mutect | 1 | 0.25 | TG/TG | 275,0 | TG/T | 297,16 | ENST00000240587.5:c.2631del | ENSP00000240587.4:p.Thr878LeufsTer27 | TSHZ3 |
| HB_D_TT | chr20 | 21396466 | C | T | Intersection | 1 | 0.25 | C/C | 199,0 | C/T | 225,153 | ENST00000351817.5:c.510G>A | ENSP00000345147.4:p.Ala170= | NKX2-4 |
| HB_D_TT | chr22 | 21772875 | C | T | Intersection | 1 | 0.25 | C/C | 52,0 | C/T | 30,13 | ENST00000215832.10:c.964G>A | ENSP00000215832.6:p.Glu322Lys | MAPK1 |
| HB_D_TT | chr22 | 43711579 | C | T | mutect | 1 | 0.25 | C/C | 148,0 | C/T | 89,6 | ENST00000262726.12:c.927G>A | ENSP00000262726.7:p.Gly309= | EFCAB6 |
| HB_E_TT | chr3 | 151430325 | CCAG | C | mutect | 1 | 0.25 | CCAG/CCAG | 214,0 | CCAG/C | 228,16 | ENST00000474524.5:c.6343_6345del | ENSP00000417235.1:p.Gln2115del | MED12L |
| HB_E_TT | chr3 | 168029853 | CCTG | C | mutect | 1 | 0.25 | CCTG/CCTG | 106,0 | CCTG/C | 186,14 | ENST00000470487.6:c.1357_1359del | ENSP00000417354.1:p.Gln453del | GOLIM4 |
| HB_E_TT | chr11 | 19879928 | AAGC | A | mutect | 1 | 0.25 | AAGC/AAGC | 247,1 | AAGC/A | 445,24 | ENST00000396087.7:c.587_589del | ENSP00000379396.3:p.Gln196del | NAV2 |
| HB_H_TT | chr6 | 1611567 | GGGC | G | mutect | 1 | 0.25 | GGGC/GGGC | 74,0 | GGGC/G/. | 63,8 | ENST00000645831.2:c.1139_1141del | ENSP00000493906.1:p.Gly380del | FOXC1 |
| HB_Q_TT | chr10 | 110125845 | C | A | mutect | 1 | 0.25 | C/C | 28,0 | C/A | 8,4 | ENST00000356080.9:c.1421C>A | ENSP00000348381.4:p.Ser474Ter | ADD3 |
| HB_Q_TT | chr17 | 44435118 | G | A | Intersection | 1 | 0.25 | G/G | 83,1 | G/A | 82,18 | ENST00000591680.6:c.295C>T | ENSP00000467556.1:p.Arg99Cys | GPATCH8 |
| HB_Q_TT | chr22 | 42214566 | AAGG | A | mutect | 1 | 0.25 | AAGG/AAGG | 151,1 | AAGG/A | 107,10 | ENST00000359486.7:c.737_739del | ENSP00000352463.3:p.Ser246del | TCF20 |
| HB_K_TT | chr3 | 41224607 | A | G | Intersection | 1 | 0.25 | A/A | 140,0 | A/G | 27,30 | ENST00000645320.1:c.95A>G | ENSP00000495360.1:p.Asp32Gly | CTNNB1 |
| HB_K_TT | chr10 | 93613172 | G | A | Intersection | 1 | 0.25 | G/G | 98,0 | G/A | 86,50 | ENST00000371447.4:c.447G>A | ENSP00000360502.3:p.Thr149= | PDE6C |
| HB_K_TT | chr11 | 7927786 | G | A | Intersection | 1 | 0.25 | G/G | 259,2 | G/A | 134,81 | ENST00000642108.1:c.877C>T | ENSP00000492919.1:p.Arg293Ter | OR10A6 |
| HB_K_TT | chr14 | 64113548 | G | C | Intersection | 1 | 0.25 | G/G | 96,0 | G/C | 56,24 | ENST00000358025.7:c.12817G>C | ENSP00000350719.3:p.Glu4273Gln | SYNE2 |
| HB_K_TT | chr14 | 100239469 | TCAC | T | mutect | 1 | 0.25 | TCAC/TCAC | 201,0 | TCAC/T | 116,7 | ENST00000262238.10:c.239_241del | ENSP00000262238.4:p.His80del | YY1 |
| HB_K_TT | chr17 | 29115407 | G | A | Intersection | 1 | 0.25 | G/G | 212,0 | G/A | 188,118 | ENST00000527372.6:c.2262C>T | ENSP00000437073.1:p.Gly754= | MYO18A |
| HB_K_TT | chr19 | 14799300 | G | A | varscan | 1 | 0.25 | G/G | 137,0 | G/A | 94,67 | ENST00000642030.1:c.837C>T | ENSP00000493026.1:p.Thr279= | OR7C1 |
| HB_K_TT | chrX | 72272693 | G | A | varscan | 1 | 0.25 | G/G | 28,0 | G/A | 22,7 | ENST00000316084.10:c.770C>T | ENSP00000362744.4:p.Ala257Val | RPS4X |
| HB_P_TT | chr4 | 84690554 | CCA | C | mutect | 1 | 0.25 | CCA/CCA | 449,0 | CCA/C | 192,11 | ENST00000295888.8:c.9313_9314del | ENSP00000295888.4:p.Trp3105GlyfsTer23 | WDFY3 |
| HB_P_TT | chr4 | 155953727 | C | G | mutect | 1 | 0.25 | C/C | 135,0 | C/G | 121,8 | ENST00000433477.4:c.121G>C | ENSP00000414904.3:p.Ala41Pro | CTSO |
| HB_P_TT | chr12 | 49039267 | C | CG | mutect | 1 | 0.25 | C/C | 431,0 | C/CG | 362,21 | ENST00000301067.11:c.8320dup | ENSP00000301067.7:p.Arg2774ProfsTer71 | KMT2D |
| HB_G_TT | chr6 | 70522575 | C | A | mutect | 1 | 0.25 | C/C | 105,0 | C/A | 49,5 | ENST00000418814.6:c.1092C>A | ENSP00000410768.2:p.Tyr364Ter | FAM135A |
| HB_G_TT | chr12 | 70353859 | G | C | mutect | 1 | 0.25 | G/G | 79,0 | G/C | 46,6 | ENST00000229195.7:c.1567G>C | ENSP00000229195.3:p.Glu523Gln | CNOT2 |
| HB_M_TT | chr15 | 49328612 | GTGA | G | mutect | 1 | 0.25 | GTGA/GTGA | 99,0 | GTGA/G | 110,9 | ENST00000299338.11:c.1480_1482del | ENSP00000299338.6:p.Ser494del | FAM227B |
| HB_C_TT | chr1 | 11779825 | G | T | Intersection | 1 | 0.25 | G/G | 133,2 | G/T | 113,19 | ENST00000433342.5:c.2747G>T | ENSP00000414909.2:p.Trp916Leu | C1ORF167 |
| HB_C_TT | chr1 | 34905129 | AC | A | mutect | 1 | 0.25 | AC/AC | 281,2 | AC/A | 275,17 | ENST00000373347.6:c.254del | ENSP00000362444.1:p.Gly85ValfsTer215 | DLGAP3 |
| HB_C_TT | chr2 | 27307804 | G | C | mutect | 1 | 0.25 | G/G | 56,0 | G/C | 62,9 | ENST00000296099.2:c.92C>G | ENSP00000296099.2:p.Ala31Gly | UCN |
| HB_C_TT | chr2 | 150487320 | T | G | Intersection | 1 | 0.25 | T/T | 207,2 | T/G | 184,27 | ENST00000375734.6:c.98A>C | ENSP00000364886.2:p.Gln33Pro | RDN3 |
| HB_C_TT | chr3 | 8985794 | G | T | Intersection | 1 | 0.25 | G/G | 185,3 | G/T | 210,40 | ENST00000383836.8:c.3025C>A | ENSP00000373347.3:p.Pro1009Thr | SRGAP3 |
| HB_C_TT | chr9 | 135696730 | C | T | Intersection | 1 | 0.25 | C/C | 1142,15 | C/T | 953,193 | ENST00000425225.2:c.543G>A | ENSP00000404438.1:p.Pro181= | SOHLH1 |
| HB_C_TT | chr9 | 137834417 | C | T | mutect | 1 | 0.25 | C/C | 1556,2 | C/T | 1,539,135 | ENST00000460843.6:c.3609C>T | ENSP00000417980.1:p.Cys1203= | EHMT1 |
| HB_C_TT | chr14 | 104166928 | C | T | Intersection | 1 | 0.25 | C/C | 244,3 | C/T | 190,53 | ENST00000423312.6:c.993C>T | ENSP00000388241.2:p.Arg331= | KIF26A |
| HB_C_TT | chrX | 40735323 | A | G | varscan | 1 | 0.25 | A/A | 55,0 | A/G | 41,11 | ENST00000324817.6:c.90T>C | ENSP00000323720.1:p.Pro30= | MED14 |
| HB_C_TT | chrX | 45053933 | C | T | Intersection | 1 | 0.25 | C/C | 71,2 | C/T | 19,19 | ENST00000377967.8:c.853C>T | ENSP00000367203.4:p.Gln285Ter | KDM6A |
| HB_C_TT | chrX | 136344840 | A | G | Intersection | 1 | 0.25 | A/A | 20,0 | A/G | 16,11 | ENST00000394143.6:c.1134A>G | ENSP00000377699.1:p.Arg378= | ADGRG4 |
| HB_I_TT | chr2 | 219540275 | C | T | mutect | 1 | 0.25 | C/C | 570,0 | C/T | 463,31 | ENST00000243776.11:c.1436G>A | ENSP00000243776.6:p.Arg479Gln | CHRF |
| HB_I_TT | chr5 | 98881296 | C | A | Intersection | 1 | 0.25 | C/C | 70,0 | C/A | 45,6 | ENST00000614616.4:c.2947G>T | ENSP00000483667.1:p.Glu983Ter | CHD1 |
| HB_I_TT | chr12 | 123686887 | G | A | Intersection | 1 | 0.25 | G/G | 84,0 | G/A | 97,12 | ENST00000303372.7:c.616G>A | ENSP00000304941.5:p.Val206Met | TCTN2 |
| HB_I_TT | chr16 | 67367218 | TCCATC | T | Intersection | 1 | 0.25 | TCCATC/TCCATC | 62,0 | TCCATC/T | 58,8 | ENST00000329956.11:c.959_963del | ENSP00000329943.6:p.His320LeufsTer13 | LRRC36 |
| HB_R_TT | chr6 | 132570440 | G | T | mutect | 1 | 0.25 | G/G | 256,0 | G/T | 200,21 | ENST00000275198.1:c.119G>T | ENSP00000275198.1:p.Gly40Val | TAAR6 |
| HB_R_TT | chr11 | 1239809 | GA | TC | mutect | 1 | 0.25 | GA/GA | 192,0 | GA/TC | 195,23 | ENST00000529681.5:c.3594_3595inv | ENSP00000436812.1:p.Lys1199Gln | MUC5B |
| HB_N_TT | chr2 | 178576172 | G | A | Intersection | 1 | 0.25 | G/G | 28,0 | G/A | 17,15 | ENST00000589042.5:c.69960C>T | ENSP00000467141.1:p.Asn23320= | TTN |
| HB_N_TT | chr11 | 47719977 | G | C | Intersection | 1 | 0.25 | G/G | 166,2 | G/C | 97,45 | ENST00000263773.10:c.2915C>G | ENSP00000263773.5:p.Ser972Ter | FNBP4 |
| HB_N_TT | chr12 | 49046336 | TGGCACAGGGCC | T | Intersection | 1 | 0.25 | TGGCACAGGGCC/TGGCACAGGGCC | 88,0 | TGGCACAGGGCC/T | 84,21 | ENST00000301067.11:c.4496_4506del | ENSP00000301067.7:p.Gly1499GlufsTer25 | KMT2D |
| HB_N_TT | chr17 | 59644449 | C | T | Intersection | 1 | 0.25 | C/C | 39,0 | C/T | 28,36 | ENST00000621829.4:c.216C>T | ENSP00000479606.1:p.Ile72= | CLTC |
| HB_N_TT | chr21 | 10577470 | G | A | Intersection | 1 | 0.25 | G/G | 204,0 | G/A | 112,19 | ENST00000618007.4:c.806G>A | ENSP00000484403.1:p.Arg269Gln | TPTE |
| HB_J_TT | chr3 | 41224606 | G | A | Intersection | 1 | 0.25 | G/G | 51,0 | G/A | 66,15 | ENST00000645320.1:c.94G>A | ENSP00000495360.1:p.Asp32Asn | CTNNB1 |
| HB_L_TT | chr2 | 241799078 | ATCC | A | mutect | 1 | 0.25 | ATCC/ATCC | 124,0 | ATCC/A | 130,10 | ENST00000192314.7:c.57_59del | ENSP00000192314.6:p.Leu20del | GAL3ST2 |
| HB_L_TT | chr4 | 15936554 | C | CT | mutect | 1 | 0.25 | C/C | 106,0 | C/CT | 92,8 | ENST00000382333.2:c.78dup | ENSP00000371770.1:p.Val27SerfsTer23 | FGFBP1 |
| HB_L_TT | chr8 | 22279559 | G | A | mutect | 1 | 0.25 | G/G | 103,0 | G/A | 83,8 | ENST00000356766.11:c.173G>A | ENSP00000349208.6:p.Ser58Asn | PIWIL2 |
| HB_L_TT | chr10 | 74105192 | G | A | mutect | 1 | 0.25 | G/G | 99,0 | G/A | 55,6 | ENST00000211998.9:c.2273G>A | ENSP00000211998.4:p.Arg758His | VCL |
| HB_L_TT | chr12 | 49026797 | A | ACAGGTC | Intersection | 1 | 0.25 | A/A | 232,0 | A/ACAGGTC | 256,37 | ENST00000301067.11:c.15163_15168dup | ENSP00000301067.7:p.Asp5055_Leu5056dup | KMT2D |
| HB_L_TT | chr12 | 71663475 | CGCT | C | mutect | 1 | 0.25 | CGCT/CGCT | 333,3 | CGCT/C | 379,21 | ENST00000378743.8:c.133_135del | ENSP00000368017.3:p.Ser45del | ZFC3H1 |
| HB_L_TT | chr16 | 2319701 | G | A | Intersection | 1 | 0.25 | G/G | 228,1 | G/A | 173,62 | ENST00000301732.10:c.753C>T | ENSP00000301732.5:p.Ile251= | ABCA3 |
| HB_L_TT | chr17 | 47848285 | G | T | Intersection | 1 | 0.25 | G/G | 178,0 | G/T | 135,48 | ENST00000536300.2:c.145C>A | ENSP00000438209.1:p.Pro49Thr | SP6 |
| HB_L_TT | chr20 | 32434498 | CG | C | Intersection | 1 | 0.25 | CG/CG | 214,0 | CG/C | 213,42 | ENST00000375687.10:c.1788del | ENSP00000364839.4:p.Ile597SerfsTer106 | ASXL1 |
| HB_L_TT | chrX | 55222708 | C | T | Intersection | 1 | 0.25 | C/C | 74,0 | C/T | 58,7 | ENST00000289619.9:c.338C>T | ENSP00000289619.5:p.Thr113Ile | PAGE5 |
| HB_L_TT | chrX | 119073513 | AGAG | A | varscan | 1 | 0.25 | AGAG/AGAG | 31,0 | AGAG/A | 24,8 | ENST00000620151.2:c.303_305del | ENSP00000488984.1:p.Glu103del | AL772284.2 |
| HB_L_TT | chrM | 10646 | G | A | varscan | 1 | 0.25 | G/G | 689,2 | G/A | 80,9 | ENST00000361335.1:c.177G>A | ENSP00000354728.1:p.Val59= | MT-ND4L |
| HB_L_TT | chrM | 10873 | T | C | varscan | 1 | 0.25 | T/T | 496,1 | T/C | 48,4 | ENST00000361381.2:c.114T>C | ENSP00000354961.2:p.Pro38= | MT-ND4 |

| Supplementary Table 5: Sanger sequencing confirmed the primer sequence. | | | |
| --- | --- | --- | --- |
| Primer name | Primer sequence | Amplicon size | TM |
| CTNNB1-F | ACTCCTCCTAATGGCTTGGT | 803 | 55.4 |
| CTNNB1-R | TGTCTCAGGGAACATAGCAG |  | 53.1 |
| CTNNB1-CXF | AAACAAGCCACCAGCAGGAA |  |  |
| CTNNB1-CXR | ATGGTAAAAGTGACATTGCT |  |  |
| KMT2D-F | GCTGTTGCCTGTTGTTGCTG | 858 | 59.9 |
| KMT2D-R | GGAGAAGCAACGGCAGGTAA |  | 60.3 |
| KMT2D-CXF1 | TGGGCTTAGGGCAGTGAGGA |  |  |
| KMT2D-F2 | GACAAACGGAGGTGGCTGAG | 491 | 60.2 |
| KMT2D-R2 | CTTCGGGTAGGTTGGGTGC |  | 60.1 |
| KMT2D-CXF2 | CACCCACAGGCTTTACCACG |  |  |
| KMT2D-F3 | TGCTGTTGCCTGTTGTTGC | 858 | 58.4 |
| KMT2D-R3 | GAGAAGCAACGGCAGGTAA |  | 56.2 |
| KMT2D-CXF3 | TGGGCTTAGGGCAGTGAGGA |  |  |

| Supplementary Table 6 Clinical data collection of multi center 81 HB patients. | | | | | | |
| --- | --- | --- | --- | --- | --- | --- |
| Reference | Sample | Sex | Age (M) | AFP (ng/mL) | Histology | PRETEXT |
| 2020,BRAZILIAN(PMID:32432034) | HB15 | F | 18 | 5,668,000 | Epithelial (embryonal) | 4 |
| 2020,BRAZILIAN(PMID:32432034) | HB16 | M | 9 | 824 | Epithelial (fetal) | 4 |
| 2020,BRAZILIAN(PMID:32432034) | HB17 | F | 36 | >400,000 | Epithelial (fetal) | 1 |
| 2020,BRAZILIAN(PMID:32432034) | HB18 | M | 9 | >200,000 | Mixed epithelial and mesenchymal | 3 |
| 2014,CHINA (PMID:24912477) | HB2 | F | 23 | >121000 | Epithelial (fetal and embryonal) | - |
| 2014,GERMANY(PMID:25135868) | HB-253 | F | 11 | >100 | Epithelial (fetal) | 4 |
| 2014,GERMANY(PMID:25135868) | HB-254 | M | 4 | >100 | Epithelial (fetal) | 2 |
| 2020,BRAZILIAN(PMID:32432034) | HB28 | M | 204 | - | Mixed epithelial and mesenchymal | 4 |
| 2014,CHINA (PMID:24912477) | HB3 | M | 13 | >121000 | Epithelial (embryonal) | - |
| 2020,BRAZILIAN(PMID:32432034) | HB30 | M | 54 | >1,000,000 | HB with HCC features | 2 |
| 2020,BRAZILIAN(PMID:32432034) | HB31 | M | 30 | 742,000 | Epithelial (fetal) | 3 |
| 2020,BRAZILIAN(PMID:32432034) | HB32 | F | 36 | 9,328,000 | Mixed epithelial and mesenchymal | 4 |
| 2020,BRAZILIAN(PMID:32432034) | HB33 | F | 1 | 28312000 | Epithelial (fetal and embryonal) | 2 |
| 2020,BRAZILIAN(PMID:32432034) | HB46 | M | 28 | >200,000 | Mixed epithelial and mesenchymal | 4 |
| 2014,CHINA (PMID:24912477) | HB5 | M | 7 | >121000 | Epithelial (fetal) | - |
| 2014,GERMANY(PMID:25135868) | HB-528 | M | 39 | >100 | - | 2 |
| 2014,GERMANY(PMID:25135868) | HB-577 | F | 38 | >100 | Epithelial (fetal) | 4 |
| 2014,CHINA (PMID:24912477) | HB6 | M | 2 | >100000 | Epithelial (embryonal) | - |
| 2014,GERMANY(PMID:25135868) | HB-612 | M | 6 | >100 | Mixed differentiation, Fetal histology | 3 |
| 2014,GERMANY(PMID:25135868) | HB-629 | F | 48 | >100 | Epithelial (fetal) | 2 |
| 2014,GERMANY(PMID:25135868) | HB-667 | M | 8 | >100 | - | 4 |
| 2014,GERMANY(PMID:25135868) | HB-678 | M | 12 | >100 | Mixed differentiation, Fetal histology | 1 |
| 2014,GERMANY(PMID:25135868) | HB-690 | M | 9 | >100 | Epithelial (fetal) | 3 |
| 2014,GERMANY(PMID:25135868) | HB-692 | M | 10 | >100 | Mixed differentiation, Fetal histology | 2 |
| 2014,GERMANY(PMID:25135868) | HB-734 | F | 33 | >100 | Epithelial (fetal) | 1 |
| 2014,GERMANY(PMID:25135868) | HB-744 | M | 6 | >100 | Mixed differentiation, Fetal histology | 3 |
| 2014,GERMANY(PMID:25135868) | HB-794 | F | 9 | >100 | Epithelial (fetal) | 2 |
| 2014,CHINA (PMID:24912477) | HB8 | F | 60 | >100000 | Epithelial (fetal) | - |
| 2014,GERMANY(PMID:25135868) | HB-804 | F | 15 | >100 | Mixed differentiation, Fetal histology | 1 |
| 2014,GERMANY(PMID:25135868) | HB-843 | M | 47 | >100 | Epithelial (fetal) | 3 |
| 2014,CHINA (PMID:24912477) | HB9 | F | 10 | 5620 | Epithelial (fetal) | - |
| 2020,CHINA (our center) | HB-C | M | 43 | 686400 | Epithelial (fetal) | 4 |
| 2020,CHINA (our center) | HB-D | M | 76 | 782230 | Epithelial (embryonal) | 3 |
| 2020,CHINA (our center) | HB-E | M | 8 | 282250 | Epithelial (fetal) | 3 |
| 2020,CHINA (our center) | HB-F | F | 17 | 420190 | Epithelial (fetal and embryonal) | 3 |
| 2020,CHINA (our center) | HB-G | M | 16 | 10223 | Mixed epithelial and mesenchymal | 4 |
| 2020,CHINA (our center) | HB-H | M | 18 | 319274 | Mixed epithelial and mesenchymal | 3 |
| 2020,CHINA (our center) | HB-I | M | 30 | 742020 | Epithelial (embryonal) | 3 |
| 2020,CHINA (our center) | HB-J | F | 8 | 330509 | Mixed epithelial and mesenchymal | 3 |
| 2020,CHINA (our center) | HB-K | M | 8 | >1210 | Epithelial (fetal) | 2 |
| 2020,CHINA (our center) | HB-L | M | 7 | 72969 | Epithelial (embryonal) | 2 |
| 2020,CHINA (our center) | HB-M | F | 9 | 5173 | Epithelial (fetal) | 2 |
| 2020,CHINA (our center) | HB-N | M | 23 | >1210 | Epithelial (embryonal) | 2 |
| 2020,CHINA (our center) | HB-O | M | 22 | 296250 | Epithelial (fetal and embryonal) | 3 |
| 2020,CHINA (our center) | HB-P | F | 15 | 123012 | Epithelial (fetal and embryonal) | 2 |
| 2020,CHINA (our center) | HB-Q | F | 12 | 5426 | Epithelial (fetal) | 1 |
| 2020,CHINA (our center) | HB-R | M | 7 | 20238 | Mixed epithelial and mesenchymal | 2 |
| 2017,USA(PMID:27775819) | TLT-001 | M | 9 | - | Epithelial (fetal, embryonal and minor small cell) | 1 |
| 2017,USA(PMID:27775819) | TLT-003 | F | 20 | - | Epithelial (mitotically active fetal and embryonal) |  |
| 2017,USA(PMID:27775819) | TLT-004 | M | 28 | - | Epithelial (fetal and embryonal) |  |
| 2017,USA(PMID:27775819) | TLT-005 | M | 5 | - | Epithelial (fetal and embryonal) | 1 |
| 2017,USA(PMID:27775819) | TLT-006 | M | 15 | - | Epithelial (fetal and embryonal) | - |
| 2017,USA(PMID:27775819) | TLT-007 | M | 20 | - | Epithelial (fetal and embryonal) | 3 |
| 2017,USA(PMID:27775819) | TLT-009 | F | 106 | - | Hepatocellular neoplasm, NOS | 3 |
| 2017,USA(PMID:27775819) | TLT-011 | F | 11 | - | Epithelial (predominantly fetal and embryonal) | 1 |
| 2017,USA(PMID:27775819) | TLT-012 | M | 11 | - | Mixed epithelial and mesenchymal | 3 |
| 2017,USA(PMID:27775819) | TLT-013 | F | 6 | - | Epithelial (fetal) | 1 |
| 2017,USA(PMID:27775819) | TLT-014 | M | 6 | - | Epithelial (mixed fetal and small cell) | - |
| 2017,USA(PMID:27775819) | TLT-015 | M | 36 | - | Epithelial (fetal and embryonal) | 1 |
| 2017,USA(PMID:27775819) | TLT-016 | M | 22 | - | Epithelial (embryonal, fetal and minor small cell) | 1 |
| 2017,USA(PMID:27775819) | TLT-017 | M | 20 | - | Epithelial (fetal) | 1 |
| 2017,USA(PMID:27775819) | TLT-018 | F | 40 | - | Epithelial (fetal and embryonal) | - |
| 2017,USA(PMID:27775819) | TLT-019 | F | 6 | - | Mixed epithelial and mesenchymal | 1 |
| 2017,USA(PMID:27775819) | TLT-021 | F | 28 | - | Mixed, predominately epithelial, small area of osteoid | 3 |
| 2017,USA(PMID:27775819) | TLT-023 | F | 12 | - | Epithelial (fetal and embryonal) | - |
| 2017,USA(PMID:27775819) | TLT-024 | M | 11 | - | Mixed epithelial and mesenchymal | 1 |
| 2017,USA(PMID:27775819) | TLT-028 | M | 4.8 | - | Epithelial (embryonal) | 1 |
| 2017,USA(PMID:27775819) | TLT-031 | M | 18 | - | Epithelial (fetal) | 1 |
| 2017,USA(PMID:27775819) | TLT-032 | M | 18 | - | Epithelial (mostly embryonal, small foci of fetal and rare small cell elements) | - |
| 2017,USA(PMID:27775819) | TLT-033 | F | 15 | - | Mixed epithelial and mesenchymal | - |
| 2017,USA(PMID:27775819) | TLT-040 | F | 8 | - | Epithelial (fetal, embryonal and small cell) | 1 |
| 2017,USA(PMID:27775819) | TLT-042 | F | 16 | - | Epithelial (fetal and embryonal) | 1 |
| 2017,USA(PMID:27775819) | TLT-044 | F | 9 | - | Epithelial (fetal and embryonal) | 1 |
| 2017,USA(PMID:27775819) | TLT-045 | M | 6 | - | Epithelial (fetal and embryonal) | 1 |
| 2017,USA(PMID:27775819) | TLT-046 | F | 27 | - | Epithelial (embryonal) | 2 |
| 2017,USA(PMID:27775819) | TLT-053 | F | 10 | - | Mixed epithelial and mesenchymal | 1 |
| 2017,USA(PMID:27775819) | TLT-056 | M | 40 | - | Epithelial | 4 |
| 2017,USA(PMID:27775819) | TLT-057 | M | 8 | - | Epithelial (fetal and blastemal. Small foci of small cell) | - |
| 2017,USA(PMID:27775819) | TLT-058 | F | 81 | - | Epithelial(embryonal, fetal, some anaplastia, telangiectatic) | - |
| 2017,USA(PMID:27775819) | TLT-059 | M | 50 | - | Epithelial (fetal) | - |
| 2017,USA(PMID:27775819) | TLT-060 | F | 18 | - | Epithelial (embryonal) | - |
