# Supplementary material for: Trans-Ancestry Mutation Landscape of Hepatoblastoma Genomes in Children
Source: Front Oncol. 2021 Apr 21;11:669560. doi: 10.3389/fonc.2021.669560 (PMC8096978; doi:10.3389/fonc.2021.669560)
Supplement: Supplementary file 5 [file Table_4.doc]

Supplementary Table 9 Differentially mutated genes in patients with hepatoblastoma (HB) of Asian, Caucasian and Hispanic origin

| Genes | Mutation type | Asian vs Caucasian | | | |  | Asian vs Hispanic | | | |  | Caucasian vs Hispanic | | | |
| --- | --- | --- | --- | --- | --- | --- | --- | --- | --- | --- | --- | --- | --- | --- | --- |
|  |  | Asian | Caucasian | χ^2^ | P |  | Asian | Hispanic | χ^2^ | P |  | Caucasian | Hispanic | χ^2^ | P |
| CTNNB1 | mutated | 5 | 17 | 3.981 | 0.046 |  | 5 | 6 | 2.085 | 0.15 |  | 17 | 6 | 0.039 | 0.84 |
|  | wild-type | 19 | 20 |  |  |  | 19 | 8 |  |  |  | 20 | 8 |  |  |
| EGFR | mutated | 0 | 0 | - | - |  | 0 | 1 | 1.761 | 0.18 |  | 0 | 1 | 2.696 | 0.10 |
|  | wild-type | 24 | 37 |  |  |  | 24 | 13 |  |  |  | 37 | 13 |  |  |
| ERBB4 | mutated | 0 | 0 | - | - |  | 0 | 1 | 1.761 | 0.18 |  | 0 | 1 | 2.696 | 0.10 |
|  | wild-type | 24 | 37 |  |  |  | 24 | 13 |  |  |  | 37 | 13 |  |  |
| CBL | mutated | 0 | 0 | - | - |  | 0 | 1 | 1.761 | 0.18 |  | 0 | 1 | 2.696 | 0.10 |
|  | wild-type | 24 | 37 |  |  |  | 24 | 13 |  |  |  | 37 | 13 |  |  |
| MAPK1 | mutated | 1 | 0 | 1.567 | 0.21 |  | 1 | 0 | 0.60 | 0.44 |  | 0 | 0 | - | - |
|  | wild-type | 23 | 37 |  |  |  | 23 | 14 |  |  |  | 37 | 14 |  |  |
| NFE2L2 | mutated | 0 | 4 | 2.777 | 0.10 |  | 0 | 0 | - | - |  | 4 | 0 | 1.642 | 0.20 |
|  | wild-type | 24 | 33 |  |  |  | 24 | 14 |  |  |  | 33 | 14 |  |  |
| ACVR2A | mutated | 0 | 1 | 0.66 | 0.42 |  | 0 | 0 | - | - |  | 1 | 0 | 0.396 | 0.53 |
|  | wild-type | 24 | 36 |  |  |  | 24 | 14 |  |  |  | 36 | 14 |  |  |
| TGFBR2 | mutated | 0 | 0 | - | - |  | 0 | 1 | 1.761 | 0.18 |  | 0 | 1 | 2.696 | 0.10 |
|  | wild-type | 24 | 37 |  |  |  | 24 | 13 |  |  |  | 37 | 13 |  |  |
| EP300 | mutated | 0 | 0 | - | - |  | 0 | 1 | 1.761 | 0.18 |  | 0 | 1 | 2.696 | 0.10 |
|  | wild-type | 24 | 37 |  |  |  | 24 | 13 |  |  |  | 37 | 13 |  |  |
| PIK3R1 | mutated | 0 | 0 | - | - |  | 0 | 1 | 1.761 | 0.18 |  | 0 | 1 | 2.696 | 0.10 |
|  | wild-type | 24 | 37 |  |  |  | 24 | 13 |  |  |  | 37 | 13 |  |  |
| TSC2 | mutated | 0 | 0 | - | - |  | 0 | 1 | 1.761 | 0.18 |  | 0 | 1 | 2.696 | 0.10 |
|  | wild-type | 24 | 37 |  |  |  | 24 | 13 |  |  |  | 37 | 13 |  |  |
| KMT2D | mutated | 4 | 1 | 2.28 | 0.05 |  | 4 | 0 | 1.90 | 0.11 |  | 1 | 0 | 0.396 | 0.53 |
|  | wild-type | 20 | 36 |  |  |  | 20 | 14 |  |  |  | 36 | 14 |  |  |

Supplementary Table 10 Differentially Gene Ontology (GO) in patients with hepatoblastoma (HB) of Asian, Caucasian and Hispanic origin

| Term | Mutation type | Asian vs Caucasian | | | |  | Asian vs Hispanic | | | |  | Caucasian vs Hispanic | | | |
| --- | --- | --- | --- | --- | --- | --- | --- | --- | --- | --- | --- | --- | --- | --- | --- |
|  |  | Asian | Caucasian | χ^2^ | P |  | Asian | Hispanic | χ^2^ | P |  | Caucasian | Hispanic | χ^2^ | P |
| GO:0005886 | mutated | 0 | 9 | 6.848 | 0.009 |  | 0 | 1 | 1.761 | 0.019 |  | 9 | 1 | 1.902 | 0.168 |
|  | wild-type | 24 | 28 |  |  |  | 24 | 13 |  |  |  | 28 | 13 |  |  |
| GO:0016477 | mutated | 0 | 0 | - | - |  | 0 | 1 | 1.761 | 0.019 |  | 0 | 1 | 2.696 | 0.10 |
|  | wild-type | 24 | 37 |  |  |  | 24 | 13 |  |  |  | 37 | 13 |  |  |

Supplementary Table 11 Differentially Pathway in patients with hepatoblastoma (HB) of Asian, Caucasian and Hispanic origin

| Pathway | Mutation type | Asian vs Caucasian | | | |  | Asian vs Hispanic | | | |  | Caucasian vs Hispanic | | | |
| --- | --- | --- | --- | --- | --- | --- | --- | --- | --- | --- | --- | --- | --- | --- | --- |
|  |  | Asian | Caucasian | χ^2^ | P |  | Asian | Hispanic | χ^2^ | P |  | Caucasian | Hispanic | χ^2^ | P |
| WNT pathway | mutated | 5 | 17 | 3.981 | 0.046 |  | 5 | 7 | 3.481 | 0.062 |  | 17 | 7 | 0.067 | 0.80 |
|  | wild-type | 19 | 20 |  |  |  | 19 | 7 |  |  |  | 20 | 7 |  |  |
| NRF2 pathway | mutated | 0 | 4 | 2.777 | 0.10 |  | 0 | 0 | - | - |  | 4 | 0 | 1.642 | 0.20 |
|  | wild-type | 24 | 33 |  |  |  | 24 | 14 |  |  |  | 33 | 14 |  |  |
| RTK/RAS pathway | mutated | 1 | 0 | 1.567 | 0.21 |  | 1 | 2 | 1.245 | 0.26 |  | 0 | 2 | 5.501 | 0.019 |
|  | wild-type | 23 | 37 |  |  |  | 23 | 12 |  |  |  | 37 | 12 |  |  |
| TGF Beta pathway | mutated | 0 | 1 | 0.66 | 0.42 |  | 0 | 1 | 1.761 | 0.19 |  | 1 | 1 | 0.532 | 0.47 |
|  | wild-type | 24 | 36 |  |  |  | 24 | 13 |  |  |  | 36 | 13 |  |  |
| NOTCH pathway | mutated | 0 | 0 | - | - |  | 0 | 1 | 1.761 | 0.19 |  | 0 | 1 | 2.696 | 0.10 |
|  | wild-type | 24 | 37 |  |  |  | 24 | 13 |  |  |  | 37 | 13 |  |  |
| PI3K Beta pathway | mutated | 0 | 0 | - | - |  | 0 | 1 | 1.761 | 0.19 |  | 0 | 1 | 2.696 | 0.10 |
|  | wild-type | 24 | 37 |  |  |  | 24 | 13 |  |  |  | 37 | 13 |  |  |
| HIPPO Beta pathway | mutated | 0 | 0 | - | - |  | 0 | 2 | 3.619 | 0.057 |  | 0 | 2 | 5.501 | 0.019 |
|  | wild-type | 24 | 37 |  |  |  | 24 | 12 |  |  |  | 37 | 12 |  |  |

Supplementary Table 12 The proportion of mutation samples in non-chemotherapy group and chemotherapy group before operation was statistically analyzed (22 Asian patients).

| Genes | Mutation type | Patients number | | | |
| --- | --- | --- | --- | --- | --- |
|  |  | non-chemotherapy group | chemotherapy group | χ^2^ | P |
| CTNNB1 | mutated | 3 | 1 | 0.512 | 0.47 |
|  | wild-type | 10 | 8 |  |  |
| KMT2D | mutated | 3 | 1 | 0.512 | 0.47 |
|  | wild-type | 10 | 8 |  |  |
| NAV2 | mutated | 0 | 1 | 1.513 | 0.22 |
|  | wild-type | 13 | 8 |  |  |
| TTN | mutated | 1 | 0 | 0.725 | 0.39 |
|  | wild-type | 12 | 9 |  |  |
| ITPR2 | mutated | 0 | 1 | 1.513 | 0.22 |
|  | wild-type | 13 | 8 |  |  |
| DLGAP3 | mutated | 0 | 1 | 1.513 | 0.22 |
|  | wild-type | 13 | 8 |  |  |
| ASXL1 | mutated | 1 | 0 | 0.725 | 0.39 |
|  | wild-type | 12 | 9 |  |  |
